# ReSurveyGermany: Vegetation-plot time-series over the past hundred years in Germany

**DOI:** 10.1038/s41597-022-01688-6

**Published:** 2022-10-19

**Authors:** Ute Jandt, Helge Bruelheide, Christian Berg, Markus Bernhardt-Römermann, Volker Blüml, Frank Bode, Jürgen Dengler, Martin Diekmann, Hartmut Dierschke, Inken Doerfler, Ute Döring, Stefan Dullinger, Werner Härdtle, Sylvia Haider, Thilo Heinken, Peter Horchler, Florian Jansen, Thomas Kudernatsch, Gisbert Kuhn, Martin Lindner, Silvia Matesanz, Katrin Metze, Stefan Meyer, Frank Müller, Norbert Müller, Tobias Naaf, Cord Peppler-Lisbach, Peter Poschlod, Christiane Roscher, Gert Rosenthal, Sabine B. Rumpf, Wolfgang Schmidt, Joachim Schrautzer, Angelika Schwabe, Peter Schwartze, Thomas Sperle, Nils Stanik, Hans-Georg Stroh, Christian Storm, Winfried Voigt, Andreas von Heßberg, Goddert von Oheimb, Eva-Rosa Wagner, Uwe Wegener, Karsten Wesche, Burghard Wittig, Monika Wulf

**Affiliations:** 1 Institute of Biology/Geobotany and Botanical Garden, Martin Luther University Halle-Wittenberg, Am Kirchtor 1, 06108 Halle, Germany; 2 German Centre for Integrative Biodiversity Research (iDiv) Halle-Jena-Leipzig, Puschstr. 4, 04103 Leipzig, Germany; 3 Karl-Franzens-Universität Graz, Institute for Biology, Holteigasse 6, 8010 Graz, Austria; 4 Institute of Ecology and Evolution, Friedrich Schiller University Jena, Dornburger Str. 159, 07743 Jena, Germany; 5 BMS - Umweltplanung, Freiheitsweg 38A, 49086 Osnabrück, Germany; 6 Abteilung Forschungsförderung, Karlsruher Institut für Technologie (KIT), Kaiserstraße 12, 76131 Karlsruhe, Germany; 7 Vegetation Ecology Group, Institute of Natural Resource Sciences (IUNR), Zurich University of Applied Sciences (ZHAW), Grüentalstr. 14, 8820 Wädenswil, Switzerland; 8 Plant Ecology, Bayreuth Center of Ecology and Environmental Research (BayCEER), Universitätsstr. 30, Bayreuth, 95447 Germany; 9 Vegetation Ecology and Conservation Biology, Institute of Ecology, FB 2, University of Bremen, Bremen, Germany; 10 Vegetation Analysis and Phytodiversity, Albrecht-von- Haller-Institute of Plant Sciences, Georg- August- University of Göttingen, Untere Karspüle 2, D-37073 Göttingen, Germany; 11 Vegetation Science and Nature Conservation Group, Institute for Biology and Environmental Sciences, University of Oldenburg, 2611 Oldenburg, Germany; 12 Auf der Wessel 47, 37085 Göttingen, Germany; 13 Department of Botany and Biodiversity Research, University of Vienna, Rennweg 14, 1030 Vienna, Austria; 14 Leuphana University of Lüneburg, Institute of Ecology, Universitätsallee 1, 21335 Lüneburg, Germany; 15 General Botany, Institute of Biochemistry and Biology, University of Potsdam, Maulbeerallee 3, 14469 Potsdam, Germany; 16 Federal Institute of Hydrology, Department Vegetation Studies, Landscape Management, Am Mainzer Tor 1, 56068 Koblenz, Germany; 17 Faculty of Agricultural and Environmental Sciences, Rostock University, Justus‐von‐Liebig‐Weg 6, 18059 Rostock, Germany; 18 Bavarian State Institute of Forestry, Hans-Carl-von-Carlowitz-Platz 1, 85354 Freising, Germany; 19 Institut für Agrarökologie und Biologischen Landbau, AG Vegetationsökologie und -monitoring, Bayerische Landesanstalt für Landwirtschaft, Lange Point 12, 85354 Freising, Germany; 20 Institute of Biology/Biology Education, Martin Luther University Halle-Wittenberg, Weinbergweg 10, 06120 Halle, Germany; 21 Universidad Rey Juan Carlos, Area de Biodiversidad y Conservación, Móstoles, Madrid, 28933 Spain; 22 Ministerium für Wissenschaft, Energie, Klimaschutz und Umwelt des Landes Sachsen-Anhalt, Leipziger Straße 58, 39112 Magdeburg, Germany; 23 Plant Ecology and Ecosystems Research, Albrecht von Haller Institute of Plant Sciences, University of Göttingen, Untere Karspüle 2, 37073 Göttingen, Germany; 24 Institute of Botany, TU Dresden, Mommsenstr. 13, 01062 Dresden, Germany; 25 Dep. Landscape Management & Restoration Ecology, Fachhochschule Erfurt, Leipzigerstr. 77, 99085 Erfurt, Germany; 26 Leibniz Centre for Agricultural Landscape Research (ZALF), Eberswalder Straße 84, 15374 Müncheberg, Germany; 27 Landscape Ecology Group, Institute for Biology and Environmental Sciences, University of Oldenburg, Carl von Ossietzky Str. 9–11, 26129 Oldenburg, Germany; 28 Ecology and Conservation Biology, Institute of Plant Sciences, Faculty of Biology and Preclinical Medicine, University of Regensburg, Universitaetsstrasse 31, 93053 Regensburg, Germany; 29 Department of Physiological Diversity, UFZ, Helmholtz Centre for Environmental Research, Puschstr. 4, 04103 Leipzig, Germany; 30 Department of Landscape and Vegetation Ecology, University of Kassel, Gottschalkstrasse 26a, 34127 Kassel, Germany; 31 University of Basel, Department of Environmental Sciences, Bernoullistrasse 32, 4056 Basel, Switzerland; 32 Department of Silviculture and Forest Ecology of the Temperate Zones, Georg-August-University Göttingen, Büsgenweg 1, D-37077 Göttingen, Germany; 33 Institute for Ecosystem Research, Kiel University, Olshausenstraße 75, 24118 Kiel, Germany; 34 Faculty of Biology, Technical University Darmstadt, Schnittspahnstraße 4, 64287 Darmstadt, Germany; 35 Biologische Station Kreis Steinfurt e.V., Bahnhofstraße 71, 49545 Tecklenburg, Germany; 36 Vogtsstr. 3, 79183 Waldkirch, Germany; 37 büro áchero Vegetation and Environmental Consulting, Friedländer Straße 17a, 37133 Friedland, Germany; 38 Fachgebiet Chemische Pflanzenökologie, Fachbereich Biologie, Technische Universität Darmstadt, Schnittspahnstr. 10, D-64287 Darmstadt, Germany; 39 Institute of Ecology and Evolution, University of Jena, Dornburger Str. 159, 07743 Jena, Germany; 40 Bayceer - Uni Bayreuth, Universitätsstr. 30, 95447 Bayreuth, Germany; 41 Technische Universität Dresden, Institute of General Ecology and Environmental Protection, Pienner Straße 7, 01737 Tharandt, Germany; 42 Engbertsheide 9, 49324 Melle, Germany; 43 Meisenweg 27, 38820 Halberstadt, Germany; 44 Botany Department, Senckenberg Museum of Natural History Görlitz, Am Museum 1, 02826 Görlitz, Germany; 45 International Institute Zittau, Technische Universität Dresden, Markt 23, 02763 Zittau, Germany; 46 Lower Saxony Water Management, Coastal Protection and Nature Conservation Agency, Betriebsstelle Lüneburg, Standort Verden, Bürgermeister Münchmeyer Str. 6, 27283 Verden, Germany; 47 Institute of Biochemistry and Biology, University of Potsdam, Maulbeerallee 3, 14469 Potsdam, Germany

**Keywords:** Biodiversity, Community ecology, Conservation biology

## Abstract

Vegetation-plot resurvey data are a main source of information on terrestrial biodiversity change, with records reaching back more than one century. Although more and more data from re-sampled plots have been published, there is not yet a comprehensive open-access dataset available for analysis. Here, we compiled and harmonised vegetation-plot resurvey data from Germany covering almost 100 years. We show the distribution of the plot data in space, time and across habitat types of the European Nature Information System (EUNIS). In addition, we include metadata on geographic location, plot size and vegetation structure. The data allow temporal biodiversity change to be assessed at the community scale, reaching back further into the past than most comparable data yet available. They also enable tracking changes in the incidence and distribution of individual species across Germany. In summary, the data come at a level of detail that holds promise for broadening our understanding of the mechanisms and drivers behind plant diversity change over the last century.

## Abstract (summary)

**Table:** 

Measurement(s)	vegetation-plot resurvey data of vascular plant species
Technology Type(s)	vegetation-plot records
Factor Type(s)	Cover of species in plots
Sample Characteristic - Organism	Vascular plant species
Sample Characteristic - Environment	Terrestrial habitats
Sample Characteristic - Location	Germany

## Background & Summary

The current biodiversity crisis threatens an estimated one million species with extinction^1^. The nature and rate of observed changes depend on the spatial scale at which they are observed^2^. At the finest scale, i.e. the local scale of plant communities, vegetation-plot records have been found to become sometimes richer, sometimes poorer in species^3^, while a considerable temporal species turnover is apparent in the majority of cases^4^.

Germany has a long tradition in resurvey studies as forest inventories were established already in the 19^th^ century^5^. However, these inventories by default only include tree species and provide no information on other growth forms, and thus, on total vascular plant diversity. In contrast, vegetation scientists carried out plot surveys, so-called relevés, since the beginning of the 20^th^ century^6^, and some of these plots have been repeatedly surveyed. Such vegetation-plot time series have mainly been collected for particular habitats, such as forests^7–19^, hedgerows^20^, wet grasslands^21–24^, mesic grasslands^25–31^, dry grasslands^24,32–37^, acid grasslands and heathlands^38–40^, alpine grasslands^41,42^, rivers^43^, riverbanks^44^, peatlands^45–48^, roadsides^49^ or arable land^50–52^. Sometimes, they were recorded to assess the changes in species composition across all communities that occur in a certain area^53–57^. So far, vegetation-plot time series have not been accessible without restrictions. In contrast, open access biodiversity time-series data, such as BioTIME^58^, comprise all different types of taxonomic groups, ranging from plants, plankton and terrestrial invertebrates to vertebrates, but include only a few vegetation-plot time series. Thus, our database closes a gap for a particular region, which is Germany.

Vegetation-plot resurvey data have been extensively used to assess biodiversity changes by means of monitoring certain vegetation types in local studies, such as managed grasslands^26^ and rivers^43^. More recently, time series have been collected across regions, exploring the contribution of local biodiversity change^3^ to that observed at broader spatial scales^1,59,60^. While these analyses often failed to detect changes in species richness^3,61,62^, they were able to relate the observed trends to changes in land use and climate^63,64^. Although these studies have compiled databases on vegetation-plot time series, they are currently not openly available. This is also the case for the current initiative of ReSurveyEurope, which collates and mobilizes vegetation-plot data with repeated measurements over time (http://euroveg.org/eva-database-re-survey-europe). Our aim is to provide a comprehensive and taxonomically standardised database of vegetation-plot time series for Germany. We confined the geographical extent to Germany because of a long tradition of German vegetation scientists carrying out temporal observations on permanent plots (e.g.^30^), the large amount of available data, our familiarity with the regional literature, and of recent initiatives to mobilize retrospective biodiversity data for trend analyses (www.idiv.de/smon).

Vegetation-plot time series differ in some fundamental ways from other biodiversity time series. Since the advent of phytosociology in the early 20th century^65,66^, vegetation surveys in Europe were carried out in a standardised way. Plot sizes of vegetation relevés can vary considerably and depend on the vegetation type considered (e.g. forest plots usually have plot sizes between 100 and 1000 m^2^, while non-forest plots mostly range from 4 to 100 m^2^ ^67^). In addition, sampling protocols might vary between studies, but they all include complete lists of species occurring at the plot at the time of sampling. In consequence, vegetation-plot records provide information on both presences and absences of species in a community. As sampling is usually done by professionals, absences of a previously occurring species in a time series strongly indicates local extinction, or vice versa, the presence of a species that had not been recorded previously is a robust indication of colonization. However, even with experts carrying out the survey, it is possible that some species may remain undetected in the record because of their phenology or taxonomic uncertainties^67^. Yet, such vegetation-plot data are much more reliable than vegetation surveys at larger scales, such as floristic grid mapping, where false absence data are common^68,69^. In contrast to time series at broader spatial scales, vegetation-plot time series contain information on species co-occurrence at scales relevant for direct biotic interactions among individuals^70^. An additional advantage of vegetation-plot records is that they report the relative abundance of species, in the case of vegetation records from Germany, typically assessed as cover values^67,71^. While species cover is very often estimated directly in per cent of ground covered by each species, there is a long tradition in vegetation science of using cover scales with distinct classes to facilitate cover estimations. There is a variety of cover scales, with different classes preferred by researchers in different countries^71,72^. The still most frequently used scale in Germany was introduced by Braun-Blanquet^6^. This scale, however, is not only based on cover, but uses the abundance of individual plants as additional criterion for species with a cover of ≤1% (classes r and +), which raises difficulties in numerical analyses^71,73^. To facilitate the estimation of cover changes in time series, Londo introduced a pure cover scale^74^, which in its original or in simplified form (e.g.^75^) became very popular in permanent plot research. It is common practice that resurvey studies use the same cover scale as in the original resurvey. Nevertheless, for a numerical comparison of changes, cover classes have to be converted into per cent values^72^, for which the Turboveg software introduced standardized transformations using the midpoints of the cover classes^76^. The cover information in vegetation-plot records allows key theories of biogeography to be tested, such as the abundance–range size relationship^77^ or the relationship between local abundance and niche breadth^78,79^. Most importantly, several vegetation-plot time series precede the onset of any other systematic plant species monitoring programme, for example the monitoring of Natura 2000 sites in Europe, which only started in 2001^80^. This is particularly important because severe biodiversity loss may have already happened in the second half of the 20^th^ century, mainly brought about by shifts in the type and intensity of land use as the consequence of technical progress and societal changes^81^. Finally, species-abundance data in plots can be linked to functional information on species^67^, which allows the interpretation of the underlying ecological drivers of the changes observed and the consequences for ecosystem functioning^82^.

Based on the data described here we analysed for the first time the dynamics of losses and gains of plant species^83^. We showed that the difference in cover changes between decreasing and increasing species results in biodiversity change even if species richness at the plot scale remains unchanged. Two mechanisms are responsible for these changes. First, losses at the plot scale were more evenly distributed among losing species than gains among winning species. Second, gains and losses in cover were concentrated in different species, resulting in a higher number of losers than winners at the spatial scale of Germany. The temporal extent of the data allowed us to demonstrate that most species losses occurred already by the 1960s, affecting mostly species from mires and spring fens, grasslands and arable land. Thus, these data already helped to shed light on the most important mechanisms underlying biodiversity change in the second half of the 20^th^ century.

## Methods

**Fig. 1:**
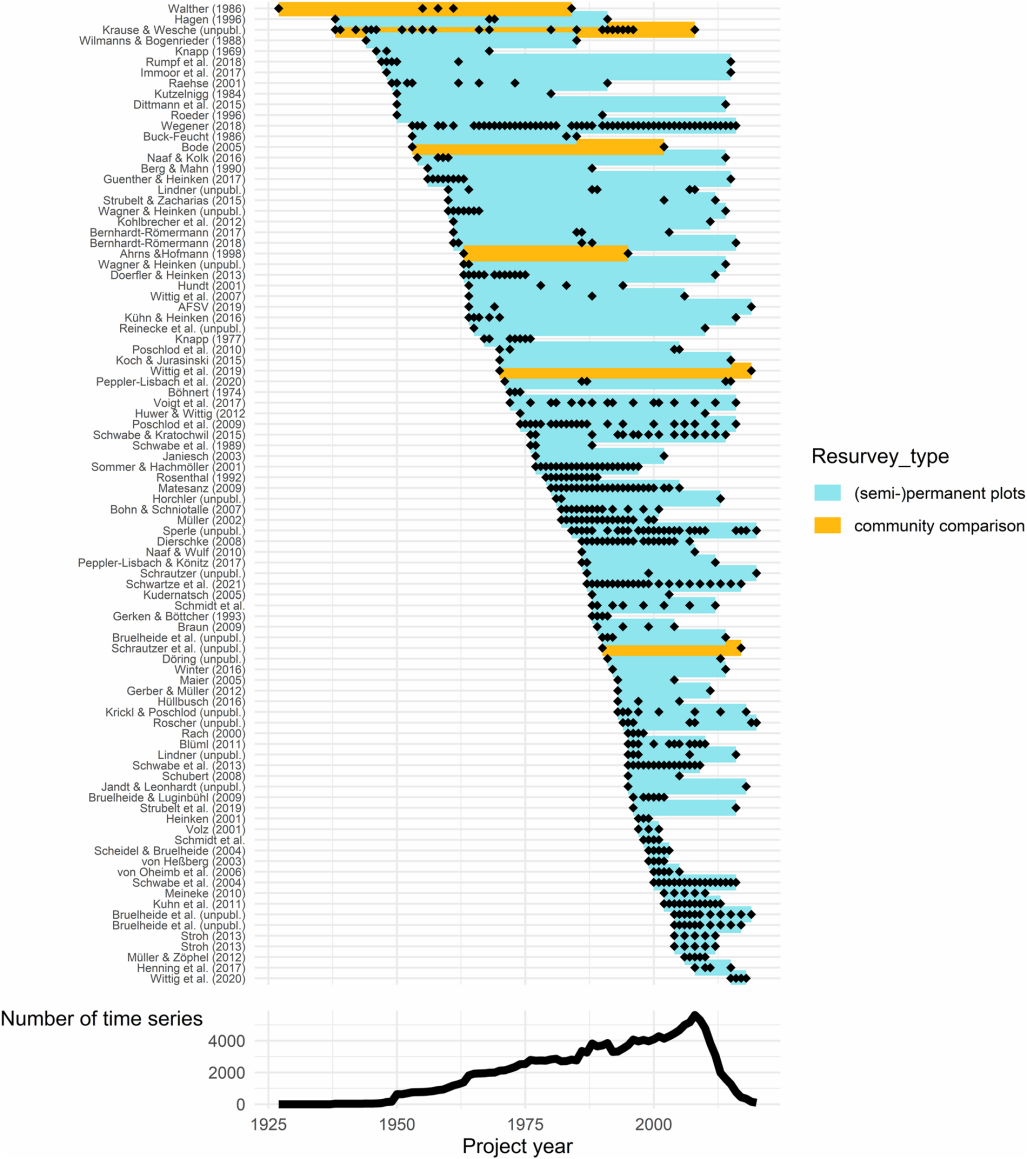
Temporal coverage of the 92 projects included in the study. The coloured lines indicate the start and the end of a project, black diamonds show in which years surveys were made. Resurvey type refers to either studies that were repeated within a particular community across a site without attempts to match plots (community comparison), or were carried out on matched plots, which were either permanently marked or retrieved from exact descriptions (semi-permanent). The lower graph shows the number of times a particular year was included in the covered time span of any of the projects. For a list of projects see Supplementary Table S1.

ReSurveyGermany is the most comprehensive compilation of repeated long-term vegetation plot records from Germany to date, including published studies as well as surveys from grey literature and nature conservation assessments. A list of all 92 projects included in the database is provided in Supplementary Table S1. A project might comprise one or several studies and focus on one or several vegetation types, but typically carried out the surveys at the same times and with the same methodology. In total, the projects comprise 1,794 vascular plant species recorded in 7,738 vegetation plots. The plots were either marked with poles or magnets (permanent) or recovered from exact descriptions, sketches or marks in high-resolution topographic maps (semi-permanent). The uncertainty of the positions varied among studies, but also within a single study as resurveyed plots might have been marked in the later surveys. If the uncertainty was provided by the author or could be estimated from topographic maps, this information was included in the PRECISION field of the header file of ReSurveyGermany (see below). In addition, there were also studies where plots were not matched in time but a set of plots at a site was compared within another set of plots at the same site in the resurvey (community comparison, Fig. 1). We only considered records with complete lists of vascular plants and information on their relative abundance, which was mostly expressed as percentage cover^84^. A further important criterion for including a study was the existence of vegetation data for at least two points in time, although the number of visits (i.e. vegetation records) per site ranged between two and 54. The time span covered by each project is shown in Fig. 1. All records were made between 1927 and 2020. In total, ReSurvey Germany comprises 23,641 vegetation-plot records and 458,311 species cover records.

**Fig. 2:**
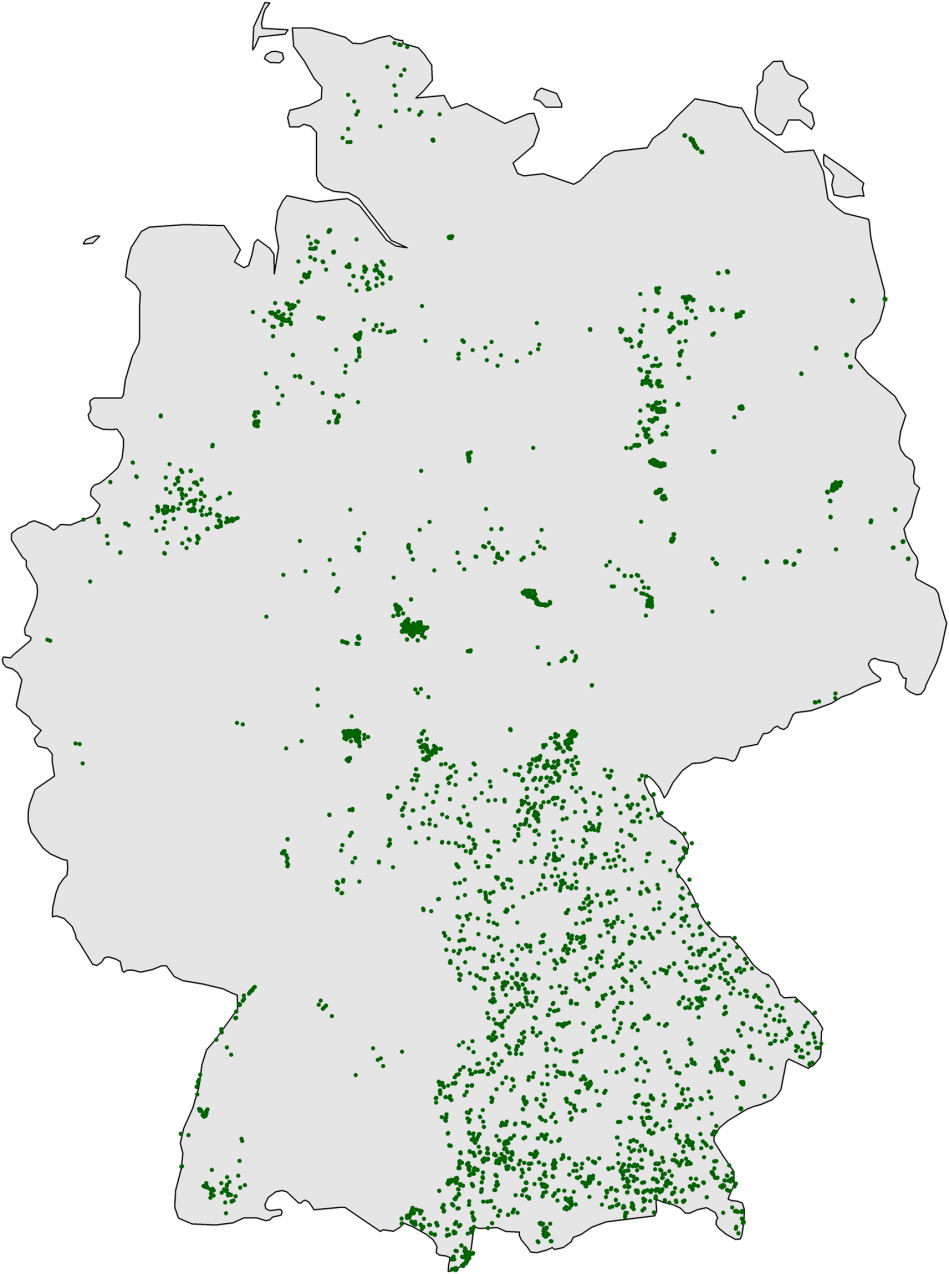
Map of all plots of all projects (n = 23,641). Note that green dots may represent one or several plots which were summarised under the same plot resurvey ID (n = 7,738). The more complete coverage of Bavaria resulted from including the grassland monitoring Bavaria which started in 2002^26^.

**Fig. 3:**
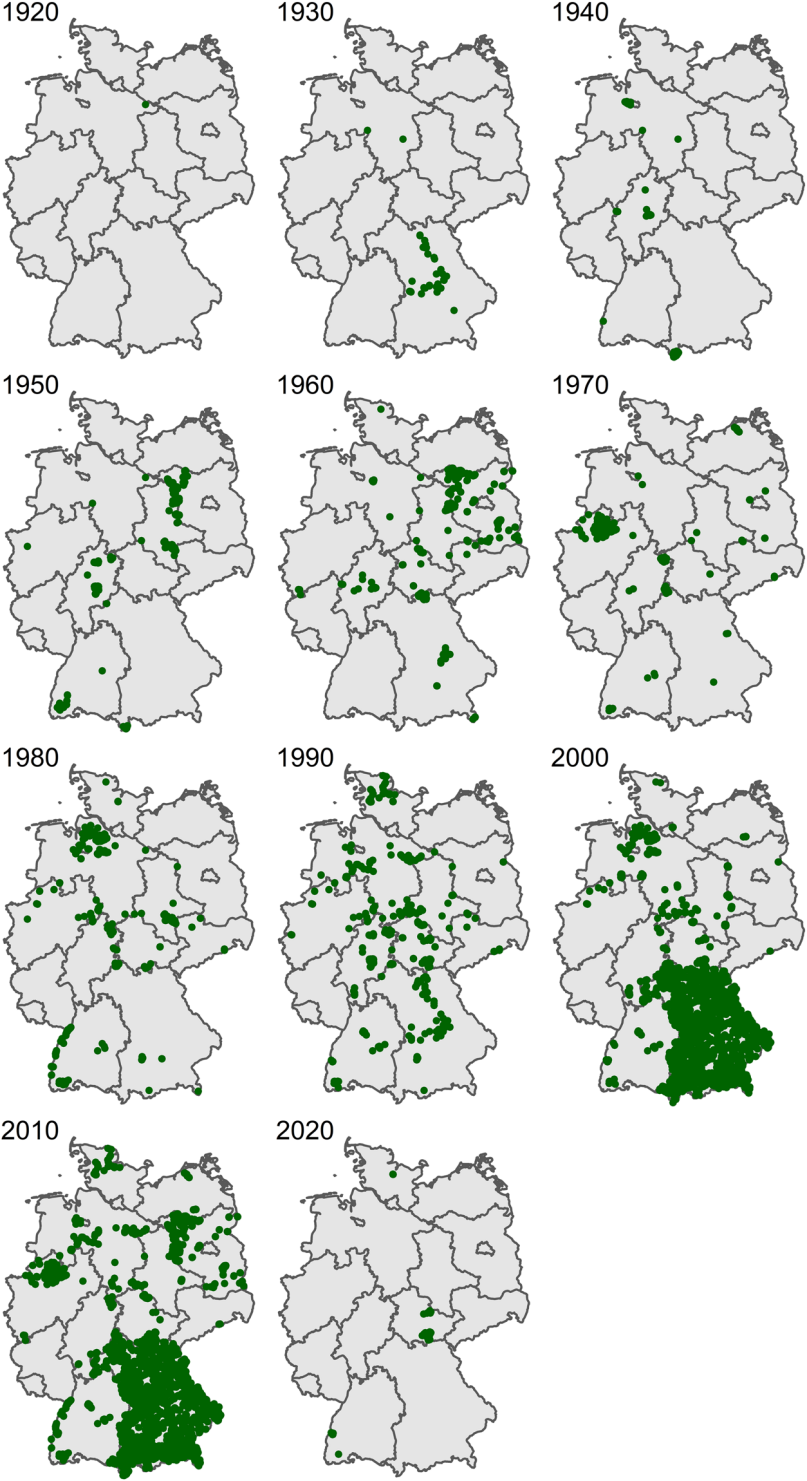
Map of plot visits by decade, with the year showing the beginning of the decade.

Plot locations are not evenly spread across Germany (Fig. 2). We assigned the individual plot locations to the grid cells of the quadrants of German ordnance maps (“MTBQ,” 0°5′ × 0°3′, approximately 5.6 km × 5.9 km in the centre of Germany), and tested whether the grid cells with vegetation-plot time-series records differed from those without observations with respect to population density, road density, urban cover, cropland cover and protected areas. Using the land cover dataset from the European Space Agency Climate Change Initiative^85^, we calculated the proportional cover of urban cover for each MTBQ. Spatial information on protected areas was obtained from GIS shapefiles provided by the German Federal Agency for Nature Conservation (Bundesamt für Naturschutz, BfN). This analysis revealed that the sampled grid cells were not representative for the whole area of Germany. As expected from other studies^86^, the sampled grid cells showed significantly higher human population densities, road densities and urban cover, while cover of cropland and the amount of protected area was lower (Table 1), which indicates that the majority of time series was made in regions with higher human pressures. The lack of spatial representativeness also becomes obvious when plotting maps of plot locations by the decade of the times when they were visited (Fig. 3).

**Table 1:** Representativeness of grid cells (“Messtischblattquadrant, MTBQ”, a quadrant of the German ordnance maps, 0°5‶ × 0°3‶) with time series.

Predictors	Population density			Road density			Urban cover			Cropland cover			Protected area		
	Estimates	CI	p	Estimates	CI	P	Estimates	CI	p	Estimates	CI	p	Estimates	CI	p
(Intercept)	30547	30128–30965	<0.001	1.59	1.55–1.63	<0.001	0.07	0.06–0.07	<0.001	0.45	0.44–0.45	<0.001	0.01	0.01–0.01	<0.001
type [unsampled]	−6686	−7447–−5925	<0.001	−0.52	−0.59–−0.45	<0.001	−0.03	−0.03–−0.03	<0.001	0.01	0.00–0.02	0.029	0.01	0.01–0.01	<0.001
Observations	11226			25303			12024			12024			29535		
R^2^/R^2^ adjusted	0.026/0.026			0.008/0.008			0.019/0.019			0.000/0.000			0.038/0.038		

The estimates were obtained from linear models comparing samples with unsampled MTBQs with respect to population density, road density, urban cover, cropland cover and protected area.

While we did not deliberately exclude certain habitat types, the data mainly consist of semi-natural to intensively managed grasslands and forests. Thus, the time series in ReSurveyGermany are biased with respect to habitat types. We assigned EUNIS habitat types to each plot record. The European Nature Information System (EUNIS) provides a comprehensive typology for the terrestrial and marine habitats of Europe^87^. Habitat types are arranged in a hierarchy, from the highest level 1 to the lowest level 4. Here, we show the assignment of plot records to level 3, which was accomplished by using the expert system EUNIS-ESy^88^ and the corresponding R code^89^. Plot records covered a total of 92 EUNIS habitat types out of the 150 ones distinguished for Germany. About 63% of the 23,641 plot records came from grasslands (level 1 EUNIS habitat R, n = 14,849), followed by forests and other wooded lands (T, n = 5,440, 23%). In contrast, arable land (V, vegetated man-made habitats), which makes up more than 36% of the land cover in Germany, was only represented by 3% (816 plot records).

## Data Records

The data of the ReSurveyGermany dataset as described above is available 10.25829/idiv.3514-0qsq70 under the terms specified by CC BY 4.0^90^.

A separate database was created for each project that contributed data, using the data-management software Turboveg 2^76^. The database is composed of two main tables, following the structure of Turboveg and common practice in vegetation science. The plot-species-abundance table contains six fields as described in Table 2. It is linked to the plot metadata (header file) through PROJECT_ID_RELEVE_NR, which is a unique Plot observation ID of a combination of PROJECT_ID (see Supplementary Table S1) and the plot observation ID (called RELEVE_NR), the name of the observed taxon (TaxonName), the vertical layer (tree layer, shrub layer, herb layer, moss layer) in which the species was observed (LAYER) and the taxon’s cover in the plot (Cover_Perc). The latter was obtained by transforming the original cover classes in per cent cover, using the midpoints of the cover classes as provided by the Turboveg software^76^. For example, the seven cover classes of the Braun-Blanquet scale^6^, r, +, 1, 2, 3, 4, 5 were transformed to 1%, 2%, 3%, 13%, 38%, 63%, 88%, respectively. The other table is the so-called header file, which holds all important plot-level information, such as plot sizes, geographic location and vegetation structure for each plot observation ID (Table 3).

**Table 2:** Data structure of the Plot-species-abundance file of ReSurveyGermany.

Field name	Type	Description
PROJECT_ID	I	Number of the resurvey project in ReSurveyGermany; see Supplementary Table S1
RELEVE_NR	I	Plot observation ID, only unique within a RS_PROJECT, usually the original plot observation ID from the underlying Turboveg 2 database
PROJECT_ID_RELEVE_NR	C	Unique Plot observation ID, by which the project’s plot-species-abundance file is linked to the header file, combination of PROJECT_ID and RELEVE_NR
LAYER	I	0: No layer, 1: Tree layer (uppermost), 2: Tree layer (middle), 3: Tree layer (lowest), 4: Shrub layer (uppermost), 5: Shrub layer (low), 6: Herb layer, 7: Juveniles, 8: Seedling (<1 year), 9: Moss layer.
TaxonName	C	Harmonized taxon name
Cover_Perc	N	Cover of the taxon in per cent

For Type: C = character, N = numeric, I = integer (n = 23,641).

**Table 3:** Data structure of the header file of ReSurveyGermany.

Field name	Type	Description	Number of NAs
RS_PROJECT	C	Unique name of the resurvey project; for the list of the 92 projects and the underlying references see Supplementary Table S1	0
PROJECT_ID	I	Number of the resurvey project in ReSurveyGermany; see Supplementary Table S1	0
RS_PLOT	C	Unique (within the site) code of the resurveyed plot; it is used to pair observations from different times recorded in the same plot; gives a unique identifier for the resurveyed plot or set of plots in time if combined with RS_PROJECT. Several plots in the same year might have the same RS_PLOT code if they have to be summarised for temporal comparisons. In these cases, they might also contain the community name.	0
RS_SITE	C	Name of the resurveyed site. For further details see LOCALITY.	0
LOCALITY	C	More detailed description of the locality of the resurveyed site, informed by the author (in German and if available)	8,499
RS_OBSERV	C	Unique code of the one-time observation; combination of RS_SITE, RS_PLOT, YEAR	0
RELEVE_NR	I	Plot observation ID, only unique within a RS_PROJECT, usually the original plot observation ID from the underlying Turboveg 2 database	0
PROJECT_ID_RELEVE_NR	C	Unique Plot observation ID, by which the project’s plot-species-abundance file is linked to the header file, combination of PROJECT_ID and RELEVE_NR	0
DATE	C	Date of the record (YYYYMMDD); the exact date if provided by the author, otherwise only the year and month or only year; if the year was not provided by the author, we took the year of the publication	0
YEAR	I	Year of the record (YYYY), extracted from DATE	0
SURF_AREA	N	Plot size [m^2^] (only stated if available)	2064
MANIPULATE	C	Binary information (Y/N) about whether the plot was part of a manipulative experiment (“Y”) or not (“N”). If “Y”, we chose the treatments representing the ambient land use. Observations with NA were to our knowledge not part of an experiment, and thus, can be treated as “N”.	16,579
MANIPTYPE	C	Shows the type of treatment in the plot manipulation (partly in German and only if available).	20,255
LAND_USE	C	Land use, informed by the authors, often identical with MANIPTYPE (mostly in German, also using the abbreviations used in the particular study, and only if available)	18,149
LOC_METHOD	C	Method of plot (re-)location, 1: Permanently marked plot isolated (i.e. somewhere within the site), 2: Marked plot in a grid (i.e. with regularly spaced neighbor plots), 3: Location with differential GPS, 4: Location with GPS, 5: Location from accurate map, 6; Location from a description, 7: Other	12,607
LOC_METH_COMMENT	C	Detailed description of the location method (if available)	20,163
LONGITUDE	N	Longitude of the plot in decimal degrees, coordinate system WGS-84; this coordinate should refer to the centre of the plot; coordinates were rounded to 2 digits of decimal degrees.	0
LATITUDE	N	Latitude of the plot in decimal degrees, rounded to 2 digits as LONGITUDE	0
PRECISION	I	Uncertainty in m, of coordinates for geographic position of plots, provided by the author or estimated if coordinates were taken from a topographic map. PRECISION refers to the true coordinates, not to those rounded to two digits.	13,034
GEO_LEV	C	Method of how the geographic location was obtained: GPS = Geographical positioning system, MTB = center of the German ordnance map, MTB_4 = center of a quadrant of the German ordnance map, POINT = all other	0
ALTITUDE	I	Elevation [m] (if available)	14,723
ASPECT	N	Compass direction of the slope in degrees [°], 0° = N, 90° = E etc. NA shows plot records either without aspect information or with aspect information when SLOPE is 0. In most cases slope aspect is simply a compass reading and has not been corrected for magnetic declination.	16,572
SLOPE	I	Inclination of the slope in degrees [°]	18,962
COUNTRY	C	DE for Germany	0
EUNIS	C	EUNIS level 3 code of the habitat, as obtained by applying the expert system EUNIS-ESy^88^ and the corresponding R code^89^.	0
COVERSCALE	C	Cover scale used for the plot record. 00 = no scale, cover estimated in per cent (%), 01 = Braun/Blanquet (old), 02 = Braun/Blanquet (new), 03 = Londo, 04 = Presence/Absence, 10 = Reichelt & Wilmanns 1973 (short), 26 = Londo (short), 29 = Londo per cent classes, 30 = Londo (modified, in project 9, Sperle *et al*. unpublished), 31 = Maas & Kohler 1983 (in project 86), 50 = Londo (modified, in project 89)	0
REFERENCE	C	Reference number in the German Vegetation Reference Database (GVRD), 6 digits referring to the bibliographic reference, found in ReSurveyGermanyReference.csv	0
YEAR_PUBL	I	Year of the publication (if available)	18,057
TABLE_NR	C	Number of the table in the original publication	12,659
TABNAME	C	Name of the table in the orginal publication	8,402
NR_IN_TAB	C	Column name in the TABLE_NR	3,789
ORIG_NR	C	Name of the plot given by the author in the original publication	10,172
ORIG_DB	C	Name of original Turboveg file, to be used internally for backtracking changes	19,700
COV_TOTAL	I	Total cover of all layers [%] (if available)	18,704
COV_TREES	I	Cover of the tree layer [%] (if available)	20,520
COV_SHRUBS	I	Cover of the shrub layer [%] (if available)	20,114
COV_HERBS	I	Cover of the herb layer [%] (if available)	11,964
COV_MOSSES	I	Cover of the moss layer (bryophytes and lichens) [%] (if available)	17,512
COV_LITTER	I	Cover of the litter layer on the ground [%] (if available)	20,786
COV_ROCK	I	Cover of the rocks on the plot surface [%] (if available)	21,697
TREE_HIGH	I	Height of the upper tree layer [m] (if available)	22,317
TREE_LOW	I	Height of the lower tree layer [m] (if available)	23,107
SHRUB_HIGH	N	Height of the upper shrub layer [m] (if available)	22,508
SHRUB_LOW	N	Height of the lower shrub layer [m] (if available)	23,478
HERB_HIGH	N	Mean height of the upper herb layer [cm] (if available)	20,311
HERB_LOW	N	Mean height of the lower herb layer [cm] (if available)	22,614
HERB_MAX	N	Maximum height of the herb layer [cm] (if available)	22,627

For Type: C = character, N = numeric, I = integer. Number of NAs = number of missing values out of the total of n = 23,641 records.

The taxon names in the plot-species-abundance table were standardised using German SL 1.3^91^. The nomenclature for vascular plants followed Wisskirchen *et al*.^92^, with additional aggregations to higher taxonomic levels according to German SL 1.3. As some authors recorded subspecies and other infraspecific taxa, species were aggregated at the species level, using the R package vegdata^93^. Some closely related species that, from our experience, are often mistaken in the field were merged at the aggregate or genus level. Species aggregates were also used when different taxon names of the same aggregate occurred in different projects, to prevent that the same taxon might appear under different taxon names. We used our own R code to merge taxon names and the notation of the ESy expert system^88^ to protocol all steps. The species harmonisation forms the first section of the ESy system and shows which taxon names were aggregated under the name of a broader taxonomic concept (Supplementary Table S2). In addition, within single projects, we used customised aggregations and segregations when the same taxa were reported with different taxonomic levels at different points in time in the same plot resurvey IDs (Supplementary Table S3). For example, in all years of a time series of a specific plot *Orchis militaris* was reported but in one year *Orchis* spec. was recorded at the genus level. Unaccounted for, such a leap between taxonomic levels within a time series would result in incorrect species change observations. To avoid losing the predominating information at the species level by aggregating all records to *Orchis*, we assumed that the taxon was also *Orchis militaris* in the particular year when only the genus level was reported. If more than one taxon occurred in previous years, we equally distributed the cover values among those taxa. This happened for example when a record was taken late in spring when the two species *Anemone nemorosa* and *A. ranunculoides* could no longer be distinguished.

The percentage cover values of the same aggregated taxon name of the same plot were merged, assuming a random overlap of their cover values and making sure that the combined cover values cannot exceed 100%^76,94^. This often resulted in cover values with decimal points and might suggest an accuracy of cover estimation that is not warranted by the original estimates. As not all projects had recorded cryptogams, we removed bryophytes and lichens in all projects, using the vegdata package in R^93^. As a result, the original list of 3,280 taxon names that included bryophytes and lichens was reduced to 1,794 taxon names of vascular plants. However, if data on lichens and bryophytes are required, they are available on request from the respective dataset custodians (see Supplementary Table S1).

The data structure of the header file of ReSurveyGermany follows the Turboveg 2 standard^76^ and in addition holds the fields of ReSurveyEurope (http://euroveg.org/eva-database-re-survey-europe) (Table 3). The fields relevant for the resurvey are RS_PROJECT, which refers to the resurvey project in Supplementary Table S1. The header field RS_SITE holds the location name of plots and allows for a local geographical scale aggregation of resurvey plots within projects. LOCALITY provides more details on the locality in German.

Within each project, the field RS_PLOT holds a plot resurvey ID that connects plot observations from different times made on the same plot. In resurveys, there are also cases, where the previously provided location was not precise enough. In these cases, resurveys often used several plots to match one previous plot, resulting in a one-to-many relationship. If a set of plots at the same site was compared with plot records from another point in time, several plot records in the same year might have the same RS_PLOT code. The unique code of the one‐time observation is a combination of RS_SITE, RS_PLOT and YEAR when the plot was surveyed (RS_OBSERV). We report the exact DATE when a record was made (if available). In addition, the field YEAR lists the year in which the plot was (re)surveyed. If available, we also report the year of the underlying publication (YEAR_PUBL).

**Fig. 4:**
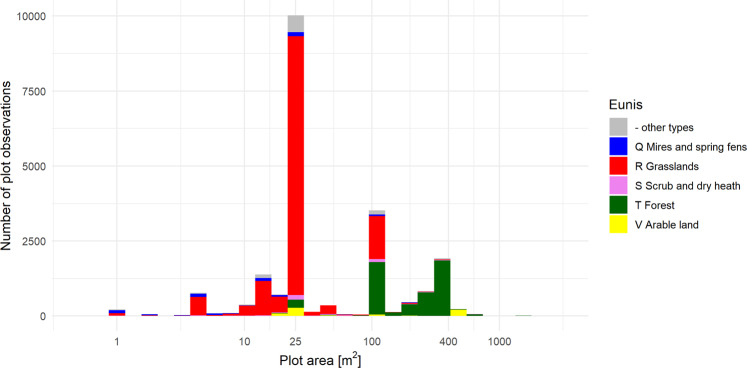
Histogram ofplot size across all records (n = 23,641). Colours show Eunis level 1 habitat types.

Plot area (SURF_AREA) ranges from 0.5 to 2500 m^2^, with 25, 100 and 400 m^2^ being the most frequently used plot sizes (Fig. 4). Plot sizes larger than 100 m^2^ were typical of forest sites (with a very few exceptions).

Geographic information is given by LONGITUDE, LATITUDE and ALTITUDE. Current monitoring programs and data protection of land owners do not allow us to provide location information at the highest available precision. In addition, some records contain occurrence data of rare and protected species. Thus, information on longitude and latitude was rounded to two decimal digits. Compared to the coordinates at highest available precision, rounding resulted in a mean uncertainty of 371 m (±138 m standard deviation), and thus, is within the somewhat limited range of accuracy provided by many custodians in the first place (see field PRECISION). If more precise coordinates are required for certain analysis we recommend to contact the respective data owners (as shown in Supplementary Table S1). Vegetation-plot time series differ with respect to the accuracy of the plot relocation during the resurvey. In the ideal case, plots are permanently marked, using poles, metal tent pegs or magnets and metal detectors to retrieve their position (shown as “01” in the LOC_METHOD field, Table 3). In other cases, plots only have exact coordinates (using GPS coordinates, “03” or “04”) or other ways of descriptions of the exact locality (such as from maps, “05”), but are not marked on the ground, which we refer to as semi-permanent plots. In addition, there is information on the cover scale used for the record, a reference to the data source (or, if published, the publication ID), including the table and column from which the data were taken.

The orientation of the plot can be taken from SLOPE (inclination) and slope ASPECT (compass directions). Vegetation structure is described by the height and cover of the different layers, ranging from tree layer to moss layer and including information on cover of litter and bare soil (if available).

Some of our projects included experimental treatments with different management of habitats (e.g. abandonment or establishment of grazing, succession and disturbance). Plots with experimental manipulation contain “Y” in the MANIPULATE) field. The type of manipulation can be taken from MANIPTYPE. When projects involved treatments that are not appropriate to assess biodiversity change, we included only the control plots^46^, plots that reflected the predominant land use at the site (e.g. mowing for a grassland to counteract natural succession)^22^, that were unfenced^95^ or were subjected to continuous grazing^96^.

## Technical Validation

As each dataset was transformed into a Turboveg 2 database^76^, a quality check was made when importing the data. This particularly applied to the taxonomical harmonization of the data, which at the stage of entering the data was adjusted to GermanSL 1.3^91^.

## Usage Notes

Users are urged to cite the original sources when using ReSurveyGermany in addition to the present paper (see Supplementary Table S1). As some of the time series will be continued, it might be useful to contact the respective data owners. As described above, the dataset cannot be considered representative of Germany’s vegetation, neither spatially, nor temporally, which is typical of vegetation-plot time series^97^. As plots were established with different objectives in different habitats at different points in time, analysis of vegetation-plot resurveys faces various methodological challenges^62^. Yet, we note that ReSurveyGermany covers about 60% of the 2,988 vascular plant species that occur in Germany (without subspecies and segregates^92^) and includes rare habitats which often harbour rare plant species. This means that even if our sites are not fully representative of the vegetation of Germany and its change over the last century, the data nevertheless can provide important insights into biodiversity change at the level of local communities and individual species.

## Supplementary information

Supplementary Table S1

Supplementary Table S2

Supplementary Table S3

## Data Availability

The R code to read the plot-species-abundance file (ReSurveyGermany.csv) and combine it with the header data (Header_ReSurveyGermany.csv) is provided on https://github.com/idiv-biodiversity/Read_ReSurveyGermany.
