# Supplementary material for: ReSurveyGermany: Vegetation-plot time-series over the past hundred years in Germany
Source: Sci Data. 2022 Oct 19;9:631. doi: 10.1038/s41597-022-01688-6 (PMC9581966; doi:10.1038/s41597-022-01688-6)
Supplement: Supplementary file 1 — Supplementary Table S1 [file 41597_2022_1688_MOESM1_ESM.docx]

Supplementary Table S1: List of all projects included in this study. PROJECT_ID: internal reference number. Reference: Either gives the original publication in which these data were used or, if in brackets, refer to a certain location. EUNIS habitat types of time series were assigned to the habitat type by using the earliest plot record that resulted in level 3 EUNIS classification^87^. The classification was based on the EUNIS-ESy expert system^88^ using the R code implementation^89^. If a project included several habitat types, the order of habitat types corresponds to decreasing numbers of plot records. Code for habitat types are ?: plots not assigned to any level 3 EUNIS habitat type, +: assigned to more than one level 3 EUNIS habitat type, A: Marine habitats, C: Inland surface waters, H: Inland sparsely vegetated habitats or devoid of vegetation, N: Coastal habitats, Q: Wetlands, R: Grasslands and lands dominated by forbs, mosses or lichens, S: Heathlands, scrub and tundra, T: Forests and other wooded land, V: Vegetated man-made habitats, including arable land. Cryp. recorded: Yes/No indicates whether the study aimed also at recording cryptogams (i.e. bryophytes or lichens) in any of the plots or in none. However, a “Yes” may be true only for some of the plots in a study and bryophytes and/or lichens have not been recorded completely. Similarly, a “No” does not exclude accidental recordings of cryptogams in some plot records. This information allows to contact the respective dataset custodians if data on lichens and bryophytes are required.

| PROJECT_ID | Project Name | Reference | EUNIS habitat type | Cryp. recorded |
| --- | --- | --- | --- | --- |
| 77 | AFSV (2019) | Arbeitsgemeinschaft Forstliche Standorts- und Vegetationskunde (AFSV) (2019) Nordwest-Eifel - Standorte, Waldgesellschaften, Nutzungen gestern und heute. Exkursionsführer der AFSV-Tagung 2019 in der Nordwesteifel. Verlag Dr. Kessel, Remagen-Oberwinter. | T17 | Yes |
| 1 | Ahrns & Hofmann (1998) | Ahrns, C. & Hofmann, G. (1998) Vegetationsdynamik und Florenwandel im ehemaligen mitteldeutschen Waldschutzgebiet "Hainich" im Intervall 1963 - 1995. Hercynia N.F. 31: 33-64. | T17 | Yes |
| 4 | Berg & Mahn (1990) | Berg, C. & Mahn, E.-G. (1990) Anthropogene Vegetationsveränderungen der Strassenrandvegetation in den letzten 30Jahren-die Glatthaferwiesen des Raumes Halle Saale. Tuexenia 10: 185-195. | R22, R | Yes |
| 2 | Bernhardt-Römermann (2017) | (Echinger Lohe) | T1F, T1E, T13, T | Yes |
| 3 | Bernhardt-Römermann (2018) | (Sonneberg) | T35, T32, T1F, T18, T17, T, S42, R57, R, ? | Yes |
| 5 | Blüml (2011) | Blüml, V. (2011) Langfristige Veränderungen von Flora und Vegetation des Grünlandes in der Dümmerniederung (Niedersachsen) unter dem Einfluss von Naturschutzmaßnahmen. Dissertation, Universität Bremen. | R55, R36, R35, R21, R, Qb, Q52, Q51 | No |
| 6 | Bode (2005) | Bode, F. (2005) Subrezenter Vegetations- und Landschaftswandel im Südschwarzwald. Dissertation, Albert-Ludwigs-Universität Freiburg. | S42 | Yes |
| 92 | Bohn & Schniotalle (2007) | Bohn, U. & Schniotalle, S. (2007) Hochmoor-, Grünland- und Waldrenaturierung im Naturschutzgebiet "Rotes Moor", Hohe Rhön 1981 - 2001: Ergebnisse 20-jähriger wissenschaftlicher Begleituntersuchungen im Rahmen und im Anschluss an ein E+E-Vorhaben des Bundes. Bundesamt für Naturschutz, Bonn. | T12, S92, R37, R35, R23!, R23, R1M, Qa, Q24, Q22, ? | Yes |
| 78 | Böhnert (1974) | Böhnert, W. (1978) Ökologische Untersuchungen auf den Kreidesandsteinhöhen der Harslebener Berge bei Quedlinburg. Naturschutz und naturkundliche Heimatforschung in den Bezirken Halle und Magdeburg 15 (2): 11-23 | S42, R | Yes |
| 7 | Braun (2009) | Braun, W. (2009) Ein Niedermoor wächst über Hochmoortorf. Ber. d. Bayerischen Botanischen Gesellschaft 79: 127-146. München. | Qb, Qa, Q25, Q24, H26a | Yes |
| 88 | Bruelheide & Luginbühl (2009) | Bruelheide, H. & Luginbühl, U. (2009) Peeking at ecosystem stability: making use of a natural disturbance experiment to analyze resistance and resilience. Ecology 90 (5): 1314-1325. | T1F, T1E, T17, T, H25 | No |
| 17 | Bruelheide et al. (unpubl.) | (Bergwiesen) | R55, R37, R35, R23!, R22, R1M, R, Q24, ? | Yes |
| 90 | Bruelheide et al. (unpubl.) | (Preuk) | T35, T1B, T, Sa | Yes |
| 91 | Bruelheide et al. (unpubl.) | (Schiessplatz) | T35, R | Yes |
| 8 | Buck-Feucht (1986) | Buck-Feucht, G. (1986) Vergleich alter und neuer Vegetationsaufnahmen im Forstbezirk Kirchheim unter Teck. Mittelungen des Vereins für forstliche Standortskunde und Forstpflanzenzüchtung (32): 43-49. | T1E, T18, T17, T13, T | Yes |
| 76 | Dierschke (2008) | Dierschke, H. (2008) Dynamik und Konstanz an naturnahen Flussufern -27 Jahre Dauerflächenuntersuchungen am Oderufer (Harzvorland). Braunschweiger Geobotanische Arbeiten 9: 119-138. | R55 | No |
| 10 | Dittmann et al. (2015) | Dittmann, T., Heinken, T. & Schmidt, M. (2018) Die Wälder von Magdeburgerforth (Fläming, Sachsen-Anhalt) – eine Wiederholungsuntersuchung nach sechs Jahrzehnten. Tuexenia 38: 11-42. | T35, T1E, T1B, T18, T17, T15, T13, T12, T | Yes |
| 11 | Doerfler & Heinken (2013) | Vegetation change of wet and moist forests in NE German nature reserves | T3M, T3J, T35, T1E, T17, T16, T15, T13, T12, T, S92, Qb, Qa, Q53, +, ? | Yes |
| 12 | Döring (unpubl.) | Feuchtwälder in den Landkreisen Diepholz und Uelzen; Wiederholungsaufnahmen von PD Dr. Wilfried Hakes [NW-FVA] im Rahmen des Forschungsvorhabens “NaLaMa-nT” erhoben | T1E, T16, T15, T13, T12, T | Yes |
| 62 | Gerber & Müller (2012) | Gerber, L. & Müller, F. (2012) Flora und Vegetation den Naturschutzgebietes Am Galgenteich Altenberg. Berichte der Arbeitsgemeinschaft sächsischer Botaniker N.F. 21: 65-123. | S42, S32, R1M, R | Yes |
| 13 | Gerken & Böttcher (unpubl.) | Abschlußbericht, unpubl. | V38, R22, R1A, R, H26a, ? | No |
| 15 | Günther et al. (2021) | Günther, K., Schmidt, M., Quitt, H. & Heinken, T. (2021): Veränderungen der Waldvegetation im Elbe-Havelwinkel von 1960 bis 2015. Tuexenia 41: 53-85. | T3M, T35, T1B, T16, T15, T13, T12, T, S92, S42 | Yes |
| 16 | Hagen (1996) | Hagen, T. (1996) Vegetationsveränderungen in Kalk-Magerrasen des Fränkischen Jura. Laufener Forschungsbericht 4. Bayerische Akademie f. Naturschutz u. Landschaftspflege, Laufen, Salzach. 218 S. | R1A, R16, R13 | Yes |
| 79 | Heinken (2001) | Heinken, A. (2001) Vegetationsentwicklung von Auengrünland nach Wiederüberflutung. Diss. Math.-Nat. Fak. Humboldt-Universität Berlin. 161 S. | R36, R22, R21, R, Qb, Q52, Q51 | Yes |
| 43 | Heinrich, Marstaller & Voigt (2012) | Eine Langzeitstudie zur Sukzession in Halbtrockenrasen - Strukturwandlung in einer Dauerbeobachtungsfläche im Naturschutzgebiet “Leutratal und Cospoth” bei Jena (Thüringen). Artenschutzreport Jena 30: 1-80. | T36, T1H, T19, T17, T, Sa, S35, R51!, R22, R1A, R16, H26a, ? | Yes |
| 68 | Henning et al. (2017) | Henning, K.; Lorenz, A.; von Oheimb, G.; Härdtle, W.; Tischew, S. Year-round cattle and horse grazing supports the restoration of abandoned, dry sandy grassland and heathland communities by supressing Calamagrostis. Journal for Nature Conservation 40: 120-130. | V38, S42, R1P, R1A, N19, N15 | No |
| 54 | von Heßberg (2003) | Von Heßberg, A. (2003) Landschafts- und Vegetationsdynamik entlang renaturierter Flussabschnitte von Obermain und Rodach. Dissertation, Universität Bayreuth. | V15, T13, S91, R55, R21, H25 | No |
| 74 | Horchler (unpubl.) | Horchler, P., Henrichfreise, A. Vollmer, I. (2013) Wiederholungsaufnahme von 54 Auenwald-Vegetationsaufnahmen am Oberrhein. | T1H, T1F, T1E, T13, T11, T, Sa, S35, R55, Q52, C23, ? | Yes |
| 19 | Hüllbusch et al. (2016) | Hüllbusch, E., Brandt, L.M., Ende, P. & Dengler, J. (2016) Little vegetation change during two decades in a dry grassland complex in the Biosphere Reserve Schorfheide-Chorin (NE Germany). Tuexenia 36: 395-412. | R22, R1P, R1B, R1A, R, N15!! | Yes |
| 20 | Hundt (2001) | Hundt, R. (2001) Ökologisch-geobotanische Untersuchungen an den mitteldeutschen Wiesengesellschaften unter besonderer Berücksichtigung ihres Wasserhaushaltes und ihrer Veränderung durch die Intensivbewirtschaftung. Mitteilungen aus dem Biosphärenreservat Rhön/Thüringen. 3. Monographie. 366 S. | V37, R55, R37, R36, R35, R23!, R22, R1M, R1A, R, Q53, Q43, + | No |
| 48 | Huwer & Wittig (2012) | unpublished data for: Huwer, A. & Wittig, R. (2012) Changes in the species composition of hedgerows. Tuexenia 32: 31-53. Göttingen. | T1E, T1B, T13, T12, T11, T, Sa, S37, ? | Yes |
| 21 | Immoor et al. (2017) | Immoor, A., Zacharias, D., Müller, J. & Diekmann, M. (2017) A re-visitation study (1948–2015) of wet grassland vegetation in the Stedinger Land near Bremen, North-western Germany. Tuexenia 37: 271-288. | R36, R35, R21, R, Qb | No |
| 23 | Jandt & Leonhardt (unpubl.) | (Kyffhäuser) | S42, R1B!, R1B, R1A, R16, R13 | Yes |
| 22 | Janiesch (2003) | Janiesch, P. (2003) Vegetationsökologische Untersuchungen in einem Erlenbruchwald im nördlichen Münsterland - 25 Jahre im Vergleich. Abhandlungen aus dem Westfälischen Museum für Naturkunde: Vegetation und Fauna in Westfalen (ed Westfälisches Museum für Naturkunde), pp. 71-80, Münster. | T15, T13 | No |
| 70 | Knapp (1969) | Knapp, R. (1969) Änderungen in der Vegetation Hessischer Gebirge in den letzten Jahrzehnten. Mitteilungen der Floristisch-Soziologischen Arbeitsgemeinschaft N.F. 14: 274-286 | R35, R22, R1M, R | Yes |
| 71 | Knapp (1977) | Knapp, R. (1977) Dauerflächen-Untersuchungen über die Einwirkung von Haustieren und Wild während trockener und feuchter Zeiten in Mesobromion- Halbtrockenrasen in Hessen. Mitteilungen der Floristisch-Soziologischen Arbeitsgemeinschaft N.F. 19: 269-274 | R1A | Yes |
| 87 | Koch & Jurasinski (2015) | Koch, M. & Jurasinski, G. (2015) Four decades of vegetation development in a percolation mire complex following intensive drainage and abandonment. Plant Ecology & Diversity 8: 49-60 | T16, T12, T, R55, R35, Qb, Qa, Q53, Q52, Q51, Q43, Q42, Q41, Q24, C35a, ? | Yes |
| 59 | Kohlbrecher et al. (2012) | unpublished data for: Kohlbrecher, C., Wesche, K., Hilbig, W., Leuschner, C., Meyer, S. (2012) Veränderungen der Segetalvegetation am Kyffhäusergebirge in den letzten 50 Jahren. Landschaftspflege und Naturschutz in Thüringen 49: 1-9. | V15 | No |
| 24 | Krause & Wesche (2011) | unpublished data for: Krause, B., Culmsee, H., Wesche, K., Bergmeier, E. & Leuschner, C. (2011) Habitat loss of floodplain meadows in north Germany since the 1950s. Biodiversity and Conservation 20 (11): 2347-2364. | R22, R21, R1P, R, C35a, A25c | No |
| 84 | Krickl & Poschlod (unpubl.) | (calcareous grasslands SW-Germany) | R1A, R16, H26a | No |
| 65 | Kudernatsch (2005) | Kudernatsch, T. (2005) Auswirkungen der globalen Erwärmung auf die Vegetation alpiner Kalk-Magerrasen im Nationalpark Berchtesgaden. Dissertation TU München, Department für Ökologie, Fachgebiet Geobotanik Weihenstephan. 151 S. | S22, R44, R43!, R | No |
| 25 | Kühn & Heinken (2017) | unpublished data for: Kühn, S.L., Heinken, T. (2017): Vegetationsveränderungen im NSG Bredower Forst im Verlauf von 50 Jahren – Analyse anhand historischer Vegetationsaufnahmen. Natursch. Landschaftspfl. Brandenbg. 26(4): 4-16. | T35, T1E, T1B, T18, T17, T, ? | Yes |
| 14 | Kuhn et al. (2011) | unpublished data for: Kuhn, G.; Heinz, S.; Meyer, F. (2011) Grünlandmonitoring Bayern, Ersterhebung der Vegetation 2002 - 2008. LfL Schriftenreihe Bayerische Landesanstalt für Landwirtschaft 3. | V38, V37, V15, V, S22, R55, R44, R37, R36, R35, R23!, R23, R22, R21, R1M, R1A, R16, R, Qb, Q53, Q52, Q51, Q43, Q41, C35d, ? | No |
| 26 | Kutzelnigg (1984) | Kutzelnigg, H. (1984) Veränderungen der Ackerwildkrautflora im Gebiet um Moers/Niederrhein seit 1950 und ihre Ursachen. Tuexenia (4): 81-102. | V37, V15 | No |
| 58 | Lindner (unpubl.) | (Hechtmoor) | T1B, T16, T, Sb, S92, S41, R, Qb, Qa, Q51, Q42, Q25, Q24, Q21, ? | Yes |
| 60 | Lindner (unpubl.) | (Süderbrarup) | R55, R52, R22, R1M, R | Yes |
| 27 | Maier (2005) | Maier, M. (2005) Untersuchung zur Entwicklung von Flora und Fauna in einem Feuchtwiesenschutzgebiet (Naturschutzgebiet Bornhorster Huntewiesen). Diplomarbeit Landschaftsökologie an der Carl von Ossietzky Universität Oldenburg. | R36, R35, Qb, Q53, Q51 | Yes |
| 28 | Matesanz (2009) | Matesanz, S., Brooker, R.W., Valladares, F. & Klotz, S. (2009) Temporal dynamics of marginal steppic vegetation over a 26-year period of substantial environmental change. Journal of Vegetation Science 20 (2): 299-310. | R16 | No |
| 55 | Meineke & Menge (2010) | Meineke, T, & Menge, K. (2010) Wirkungskontrolle zum PROFIL Kooperationsprogramm Naturschutz, Teilbereich Besondere Biotoptypen im FFH-Gebiet Bergwiesen und Wolfsbachtal bei Hohegeiß.  Im Auftrag des Niedersächsischen Landesbetriebs für Wasserwirtschaft Küsten- und Naturschutz, Hannover. | R23!, R21, R | No |
| 63 | Müller & Zöphel (2012) | Müller, F. & Zöphel, B. (2012) Bestandssituation, Biologie und Ökologie von Gentianella lutescens im Osterzgebirge. Berichte der Arbeitsgemeinschaft sächsischer Botaniker N.F. 21: 139-184 | R23! | Yes |
| 29 | Müller (2002) | Müller, N. (2002) Auswertung der Langzeituntersuchungen von Dauerflächen im Augsburger Stadtgebiet zur Renaturierung von Lechhaiden. Ber. Bayer. Landesamt Umweltschutz (Hrsg.): 97 S. | V38, V, R55, R37, R22, R1A, R16, N15, H26a | No |
| 30 | Naaf & Kolk (2016) | unpublished data for: Naaf, T. & Kolk, J. (2016) Initial site conditions and interactions between multiple drivers determine herb-layer changes over five decades in temperate forests. For Ecol Manag 366: 153-165. | T1H, T1E, T1B, T18, T17, T15, T13, T12, T, Q51, ? | Yes |
| 31 | Naaf & Wulf (2010) | Naaf, T. & Wulf, M. (2010) unpublished data for: Habitat specialists and generalists drive homogenization and differentiation of temperate forest plant communities at the regional scale. Biol Conserv 143: 848-855. | T1F, T1E, T17, T13, T12, T, Sa | No |
| 32 | Peppler-Lisbach & Könitz (2017) | Peppler-Lisbach, C. & Könitz, N. (2017): Vegetationsveränderungen in Borstgrasrasen des Werra-Meißner-Gebietes (Hessen, Niedersachsen) nach 25 Jahren – Tuexenia 37: 201-228. | S42, R37, R22, R21, R1M, R | Yes |
| 44 | Peppler‐Lisbach et al. (2020) | Peppler‐Lisbach, C, Stanik, N, Könitz, N, Rosenthal, G. (2020) Long‐term vegetation changes in *Nardus* grasslands indicate eutrophication, recovery from acidification, and management change as the main drivers. Applied Vegetation Science 23: 508-521. https://doi.org/10.1111/avsc.12513 | Sb, Sa, S42, R37, R35, R23!, R23, R22, R1M, R | Yes |
| 83 | Poschlod et al. (2009) | unpublished data for: Poschlod, P., Schreiber, K.-F., Mitlacher, K., Römermann, C. & Bernhardt-Römermann, M. (2009): Entwicklung der Vegetation und ihre naturschutzfachliche Bewertung. In: Schreiber, K.-F., Brauckmann, H.-J., Broll, G., Krebs, S. & Poschlod, P. (Hrsg.): Landschaftspflege und Naturschutz im Extensivgrünland. 30 Jahre Offenhaltungsversuche Baden-Württemberg. – Naturschutz-Spectrum Themen 97: 243-288. | T1E, R22, R21, R1M, R1A, N19 | No |
| 85 | Poschlod et al. (2010) | unpublished data for: Poschlod, P., Kos, M., Roauer, S., Seemann, A., Wiesmann, O., Zeltner, G., Kohler, A. (2006) Long-term monitoring in rivers of south Germany since the 1970ies - macrophytes as indicators for the assessment of water quality. In: Müller, F., Baessler, C., Schubert, H. & Klotz, S. (Eds.): Long-term ecological research. Between Theory and Application. Berlin: Springer, pp. 189-199. | Qb, Q52, Q51, C23, C22b, ? | Yes |
| 64 | Rach (2000) | Rach, C. (2000) Charakterisierung von Renaturierungsprozessen in Bruchwäldern - Ökologische Untersuchungen in zwei Landschaftsräumen Nordwestdeutschlands. Dissertation Fachbereich Biologie, Geo- und Umweltwissenschaften Universität Oldenburg. 201 S. | T15, T12, T | Yes |
| 36 | Raehse (2001) | Raehse, S. (2001) Veränderungen der hessischen Grünlandvegetation seit Beginn der 50er Jahre am Beispiel ausgewählter Tal- und Bergregionen Nord- und Mittelhessens. Kassel, University Press GmbH. 222 S. | V15, S42, R55, R37, R36, R35, R22, R21, R1M, R1A, R, Qb, Q53, Q51, N19 | Yes |
| 37 | Reinecke et al. unpubl. | unpublished data for: Reinecke, J., Klemm, G., Heinken, T. (2014): Vegetation change and homogenization of species composition in temperate nutrient-deficient Scots pine forests after 45 yr. J. Veg. Sci. 25: 113-121. | T35, T1B, T, S42, R54, ? | Yes |
| 80 | Roeder et al. (1996) | Röder, H., Fischer, A., Klöck, W. (1996) Waldentwicklung auf Quasi-Dauerflächen im Luzulo-Fagetum der Buntsandsteinrhön (Forstamt Mittelsinn) zwischen 1950 und 1990. Forstw. CB1. 115, 321-335 | T35, T1H, T18, T, S42, R, ? | Yes |
| 86 | Roscher (unpubl.) | (East Thuringia) | R51!, R22, R1A, R16, R | Yes |
| 38 | Rosenthal (1992) | Rosenthal, G. (1992) Erhaltung und Regeneration von Feuchtwiesen. Vegetationsökologische Untersuchungen auf Dauerflächen. Diss. Bot. 182: 1-283. Berlin, Stuttgart. | V15, R55, R36, R35, R, Q51 | No |
| 67 | Rumpf et al. (2018) | unpublished data for: Rumpf, S., Hülber, K., Klonner, G, Moser, D., Schütz, M., ;Wessely, J., Willner, W., Zimmermann, N., Dullinger, S. (2018) Range dynamics of mountain plants decrease with elevation. PNAS 115(8):1848-1853. | Sb, S42, S22, R56, R55, R44, R43, R41, R23!, R21, R, H32c, H26b, H24, H23 | No |
| 39 | Scheidel & Bruelheide (2004) | Scheidel, U. & Bruelheide, H. (2004) Versuche zur Beweidung von Bergwiesen im Harz. Hercynia N.F. 37: 87-101 | S32, R35, R, ? | Yes |
| 33 | Schmidt et al. | Garbitz, D. (1990): Vegetation und Standortsbedingungen im Naturwald "Staufenberg". Dipl.-Arb. Syst.-Geobot. Institut, Universität Göttingen.  Melcher. S. (1999): Flora und Vegetation im Naturwald "Großer Staufenberg" (Forstamt Walkenried, Revier Staufenberg). Dipl.-Arb. Institut f. Waldbau, Abt. I, Universität Göttingen.  Mölder, A., Streit, M., Schmidt, W. (2014): When beech strikes back: How strict nature conservation reduces herb-layer diversity and productivity in Central European deciduous forests. Forest Ecology and Management 319: 51-61.  Kohls, K. (1994): Geobotanische Untersuchungen in Wäldern des Forstamtes Sellhorn (Lüneburger Heide). Dipl.-Arb. Syst.-Geobot. Institut, Universität Göttingen.  Albrecht, B. (2000): Vegetationskundliche Untersuchungen im Naturwaldreservat "Meninger Holz" unter besonderer Berücksichtigung der Vegetationsentwicklung. Dipl.-Arb. FG Naturschutz, FB Biologie, Universität Hamburg.  Happe, E. (1995): Vegetation und Standortsverhältnisse im Naturwald und Naturschutzgebiet "Totenberg" (Bramwald).Dipl.-Arb. Institut f. Waldbau, Abt. I, Universität Göttingen.  Fischer, C., Parth, A., Schmidt, W. (2009): Vegetationsdynamik in Buchen-Naturwäldern. Ein Vergleich aus Süd-Niedersachsen. Hercynia 42: 45-68. | T3M, T35, T1F, T1E, T18, T17, T, Sa, +, ? | Yes |
| 34 | Schmidt et al. | Kompa T., Schmidt, W. (2005): Buchenwald-Sukzession nach Windwurf auf Zechstein-Standorten des südwestlichen Harzvorlandes. Hercynia N.F. 38: 233-261.  Schmidt, W. (2002): Die Naturschutzgebiete Hainholz und Staufenberg am Harzrand – Sukzessionsforschung in Buchenwäldern ohne Bewirtschaftung. Tuexenia 22: 151-213.  Schmidt, W., Heinrichs, S. (2012) 13 Jahre nach dem Sturm - Vegetationsentwicklung im Buchen-Naturwald "Königsbuche" (südwestliches Harzvorland, Niedersachsen). Hercynia 45: 81-110. | V39, T1F, T1E, T18, T17, T13, T12, T, Sa, S32, R57, R55, R, ? | Yes |
| 73 | Schrautzer (unpubl.) | Resurvey Eidertal | R55, R35, Q53, Q52 | Yes |
| 72 | Schrautzer et al. (unpubl.) | Resurvey of Härdtle - Beckmann | T17, T13, T12, T | Yes |
| 40 | Schubert (2008) | Schubert, R. (2008) Vegetationsdynamik in einigen Naturschutzgebieten Sachsen-Anhalts. Mitteilungen florist. Kart. Sachsen-Anhalt (Halle 2008) 13: 53-75. | V38, T, S42, S38, R55, R35, R22, R1A, R, Q51, ? | Yes |
| 42 | Schwabe & Kratochwil (2015) | Schwabe, A, & Kratochwil, A. (2015) Pflanzensoziologische Dauerflächen-Untersuchungen im Bannwald "Flüh" (Südschwarzwald) unter besonderer Berücksichtigung der Weidfeld-Sukzession. standort.wald 49: 5-49 | T18, T, S42, R52 | No |
| 41 | Schwabe et al. (1989) | Schwabe, A., Kratochwil, A. & Bammert, J. (1989) Sukzessionsprozesse im aufgelassenen Weidfeld-Gebiet des "Bannwald Flüh" (Südschwarzwald) 1976-1988 - Mit einer vergleichenden Betrachtung statistischer Auswertungsmethoden. Tuexenia 9: 351-370. Göttingen. | S42, R52, R1M, R, ? | Yes |
| 89 | Schwabe et al. (2004) | (Grundaufnahmen) unpublished data for: Schwabe, A.; Zehm, A., Nobis, M., Storm, C., Suess, K. (2004) Auswirkungen von Schaf-Erstbeweidung auf die Vegetation primär basenreicher Sand-Ökosysteme. Ber. NNA 1:/2004: 39-54. | V38, V37, V34, V, R1P, R1B, R1A, R13, R, N15!! | Yes |
| 57 | Schwabe et al. (2013) | unpublished data for Schwabe, A., Suess, K., Storm, C. (2013) What are the long-term effects of livestock grazing in steppic sandy grassland with high conservation value? Results from a 12-year field study. Tuexenia 33: 189-212. Göttingen. | V38, R1P, R1B, R1A, R16, R13, R11, R | Yes |
| 69 | Schwartze et al. 2021 | Schwartze, P., Birkner, L., Velbert, F. & Hölzel, N. (2021) Vielfalt durch extensive Grünlandnutzung. – 30 Jahre Dauermonitoring auf unterschiedlich bewirtschafteten Feuchtgrünlandflächen. Natur in NRW, 1/2021, 16-21. Paderborn. part of unpublished data for: Poptcheva, K., Schwartze, P., Vogel, A., Kleinebecker, T. & Hölzel, N. (2009) Changes in wet meadow vegetation after 20 years of different management in a field experiment (North-West Germany). Agriculture, Ecosystems & Environment, 134 (1-2), 108–114. | R55, R37, R36, R35, R21, R, Q53 | Yes |
| 61 | Sommer & Hachmöller (2001) | Sommer, S. & Hachmöller, B. (2001) Auswertung der Vegetationsaufnahmen von Dauerbeobachtungsflächen auf Bergwiesen im NSG Oelsen bei varierter Mahd im Vergleich zur Brache. Berichte der Arbeitsgemeinschaft sächsischer Botaniker N.F. 18: 99-135 | R23! | No |
| 9 | Sperle (unpubl.) |  | V39, V11, T3K, T12, Sb, S42, S38, R56, R55, R37, R35, R22, R1A, R, Qb, Qa, Q51, Q42, Q41, Q25, Q24, ? | Yes |
| 45 | Stroh (2013) | Stroh, H.-G. (2013) Wiederholung der vegetationskundlichen Erfassungen an den Dauerbeobachtungsflächen der Kalkmagerrasen des Altendorfer Berges. Bericht für das Jahr 2012. Im Auftrag des Niedersächsischen Landesbetriebs für Wasserwirtschaft Küsten- und Naturschutz, Hannover. | R1A, ? | Yes |
| 46 | Stroh (2013) | Stroh, H.-G. (2013) Wiederholung der vegetationskundlichen Erfassungen an den Dauerbeobachtungsflächen der Kalkmagerrasen der Weper und des Gladebergs. Zwischenbericht für das Jahr 2012. Im Auftrag des Niedersächsischen Landesbetriebs für Wasserwirtschaft Küsten- und Naturschutz, Hannover. | Sa, R1A, ? | Yes |
| 47 | Strubelt & Zacharias (2015) | Strubelt, I., Diekmann, M. & Zacharias, D. (2015) Langzeitmonitoring der Vegetation über 52 Jahre im Hartholzauenwald (Querco-Ulmetum minoris Issler 1924) im Haseder Busch (Landkreis Hildesheim). Braunschweiger Geobotanische Arbeiten 11: 173-247 | T3M, T1F, T1E, T17, T13, T | No |
| 66 | Strubelt et al. (2019) | Strubelt, I., Diekmann, M., Peppler-Lisbach, C., Gerken, A. & Zacharias, D. (2019) Vegetation changes in the Hasbruch forest nature reserve (NW Germany) depend on management and habitat type. Forest Ecology and Management, 444, 78–88. | T1E, T1B, T18, T17, T13, T | Yes |
| 56 | Volz (2001) | Volz, H. (2001) Vegetationskundliches Monitoring im NSG Lange Rhön Gebiet Leitgraben. Bearbeitungszeitraum 2001. Gutachten Umweltplanung Volz, Giessen, 62 S. | R57, R35 | No |
| 35 | von Oheimb et al. (2006) | v. Oheimb, G., Eischeid, I., Finck, P., Grell, H., Härdtle, W., Mierwald, U., Riecken, U., Sandkühler, J. (2006) Halboffene Weidelandschaft Höltigbaum. Perspektiven für den Erhalt und die naturverträgliche Nutzung von Offenlandlebensräumen. Naturschutz und Biologische Vielfalt 36: 1-280. Landwirschaftsverlag Münster. | V39, T13, T, R55, R52, R36, R35, R22, R21, R1P, R1M, R, Qb, Q53, Q52, N15, C35a, ? | Yes |
| 50 | Wagner & Heinken (unpubl.) |  | T3M, T35, T1H, T1E, T1B, T18, T13, T, S42, R, ? | Yes |
| 51 | Wagner & Heinken (unpubl.) |  | T35, T1F, T1E, T1B, T18, T17, T13, T12, T, R55, R51, R1M, R, ? | Yes |
| 49 | Walther (1986) | Walther, K. (1986) Die Vegetation des Maujahn 1984. Wiederholung der vegetationskundlichen Untersuchung eines wendländischen Moores. Tuexenia 6: 145-193. Göttingen. | T1B, R37, R35, R1P, Q51 | Yes |
| 52 | Wegener (2018) | Wegener, U. (2018) Vegetationswandel des Berggrünlands nach Untersuchungen von 1954 bis 2016. Wege zur Erhaltung der Bergwiesen. Abh. und Ber. aus dem Museum Heineanum 11: 35-101. | R23 | No |
| 53 | Wilmanns & Bogenrieder (1988) | Wilmanns, O. & Bogenrieder, A. (1986) Veränderungen der Buchenwälder des Kaiserstuhls im Laufe von vier Jahrzehnten und ihre Interpretation - pflanzensoziologische Tabellen als Dokumente. Abhandlungen aus dem westfälischen Museum für Naturkunde 48(2): 55-80 | T18, T, ? | Yes |
| 81 | Winter (unpubl.) | Winter, R. (2016) Flora und Vegetationsentwicklung der Sukzessionsfläche "Wildnis am Bunker Valentin" in der Bremer Weseraue bei Farge. Bachelorthesis Hochschule Bremen, Studiengang Technische und angewandte Biologie, 61 S. | T, S42, Q51 | No |
| 18 | Wittig et al. (2007) | unpublished data for: Wittig, B., Waldmann, T., Diekmann, M. (2007) Veränderungen der Grünlandvegetation im Holtumer Moor über vier Jahrzehnte. Hercynia N.F. 40: 285-300. | V38, V37, V15, V11, V, T16, T15, T13, T12, S92, S41, R55, R52, R37, R36, R35, R22, R21, R, Qa, Q53, Q51, Q22 | Yes |
| 75 | Wittig et al. (2019) | Wittig, B., Müller, J., Mahnke-Ritoff, A. (2019) Talauen-Glatthaferwiesen im Verdener Wesertal (Niedersachsen). Tuexenia 39: 249-265. Göttingen | R22 | Yes |
| 82 | Wittig et al. (2020) | Wittig, B., Müller, J., Quast, R., Miehlich, H. (2020) Arnica montana in Calluna-Heiden auf dem Schießplatz Unterlüß (Niedersachsen). Tuexenia 40: 131-146. Göttingen. | S42, R1M | Yes |
