# Supplementary material for: ReSurveyGermany: Vegetation-plot time-series over the past hundred years in Germany
Source: Sci Data. 2022 Oct 19;9:631. doi: 10.1038/s41597-022-01688-6 (PMC9581966; doi:10.1038/s41597-022-01688-6)
Supplement: Supplementary file 2 — Supplementary Table S2 [file 41597_2022_1688_MOESM2_ESM.docx]

Supplementary Table S2: List of all taxa resulting from the harmonisation across all projects. The format of the list follows the rules of the ESy system^88^. The taxon names that were aggregated below a broader concept name are indented using five blanks. The number to the right shows the German SL 1.3^91^ number for each taxon.

Achillea atrata agg. 18

Achillea atrata 19

Achillea atrata agg. 18

Achillea millefolium agg. 27

Achillea millefolium 31

Achillea millefolium agg. 27

Achillea millefolium subsp. collina 20096

Achillea millefolium subsp. millefolium 32

Achillea pannonica 34

Achillea setacea 36

Acinos arvensis 49

Acinos arvensis 49

Calamintha acinos 976

Satureja acinos 23760

Aconitum lycoctonum 14242

Aconitum lycoctonum 14242

Aconitum lycoctonum subsp. vulparia 20209

Aconitum vulparia 68

Adonis aestivalis 76

Adonis aestivalis 76

Adonis aestivalis var. citrinus 27247

Agrimonia eupatoria 99

Agrimonia eupatoria 99

Agrimonia eupatoria subsp. eupatoria 100

Agrostis canina agg. 120

Agrostis canina 121

Agrostis coarctata 20180

Agrostis stricta 122

Agrostis vinealis 20684

Agrostis capillaris 20178

Agrostis capillaris 20178

Agrostis capillaris subsp. oreophila 7086

Agrostis tenuis 130

Agrostis vulgaris 20179

Agrostis stolonifera agg. 127

Agrostis alba var. stolonifera 27181

Agrostis gigantea 128

Agrostis stolonifera 129

Agrostis stolonifera agg. 127

Agrostis stolonifera subsp. stolonifera 6550

Aira caryophyllea 26920

Aira caryophyllea 26920

Aira caryophyllea subsp. caryophyllea 20218

Alchemilla conjuncta agg. 154

Alchemilla conjuncta agg. 154

Alchemilla hoppeana 166

Alchemilla nitida 10027

Alchemilla fissa agg. 177

Alchemilla fallax 179

Alchemilla fissa 180

Alchemilla fissa agg. 177

Alchemilla hybrida agg. 190

Alchemilla flabellata 194

Alchemilla glaucescens 195

Alchemilla hybrida agg. 190

Alchemilla mollis 200

Alchemilla acutiloba var. mollis 20237

Alchemilla vulgaris agg. 209

Alchemilla acutiloba var. stellata 11694

Alchemilla crinita 219

Alchemilla glabra 235

Alchemilla micans 20278

Alchemilla monticola 245

Alchemilla subcrenata 265

Alchemilla vulgaris 26467

Alchemilla vulgaris agg. 209

Alchemilla vulgaris auct. 20289

Alchemilla xanthochlora 273

Alisma plantago-aquatica agg. 275

Alisma gramineum 276

Alisma lanceolatum 277

Alisma plantago-aquatica 278

Alisma plantago-aquatica agg. 275

Allium scorodoprasum 12756

Allium rotundum 310

Allium scorodoprasum 12756

Allium scorodoprasum subsp. rotundum 20326

Allium senescens 6583

Allium montanum 294

Allium senescens 6583

Allium senescens subsp. montanum 20321

Allium senescens var. montanum 12712

Alopecurus pratensis agg. 334

Alopecurus pratensis 336

Alopecurus pratensis agg. 334

Alyssum alyssoides 345

Alyssum alyssoides 345

Alyssum calycinum 20351

Alyssum montanum 350

Alyssum montanum 350

Alyssum montanum subsp. gmelinii 26457

Alyssum montanum subsp. montanum 26456

Anagallis arvensis 393

Anagallis arvensis 393

Anagallis arvensis subsp. arvensis 394

Anemone narcissiflora 434

Anemonastrum narcissiflorum 20423

Anemone narcissiflora 434

Anthoxanthum aristatum 20071

Anthoxanthum aristatum 20071

Anthoxanthum puelii 466

Anthoxanthum odoratum agg. 463

Anthoxanthum alpinum 464

Anthoxanthum odoratum 465

Anthoxanthum odoratum agg. 463

Anthriscus sylvestris agg. 469

Anthriscus sylvestris 473

Anthriscus sylvestris agg. 469

Anthriscus sylvestris subsp. alpestris 20460

Anthriscus sylvestris subsp. sylvestris 20463

Anthyllis vulneraria 477

Anthyllis vulneraria 477

Anthyllis vulneraria subsp. carpatica 479

Anthyllis vulneraria subsp. pseudovulneraria 14783

Aphanes inexspectata 494

Aphanes inexspectata 494

Aphanes microcarpa 6554

Aquilegia vulgaris agg. 507

Aquilegia atrata 508

Aquilegia vulgaris 510

Arabis bellidifolia 20511

Arabis bellidifolia subsp. bellidifolia 20512

Arabis glabra 520

Arabis glabra 520

Turritis glabra 24405

Arabis hirsuta agg. 521

Arabis hirsuta 523

Arabis nemorensis 20072

Arctium minus 13702

Arctium minus 13702

Arctium minus agg. 547

Lappa communis 14348

Arenaria serpyllifolia agg. 563

Arenaria serpyllifolia 13703

Arenaria serpyllifolia agg. 563

Armeria maritima 581

Armeria elongata 577

Armeria maritima 581

Armeria maritima agg. 575

Armeria maritima subsp. elongata 20584

Armeria maritima var. elongata 20590

Armeria vulgaris 26026

Artemisia campestris agg. 594

Artemisia campestris 596

Artemisia campestris agg. 594

Artemisia campestris subsp. campestris 6466

Artemisia vulgaris agg. 616

Artemisia vulgaris 618

Artemisia vulgaris agg. 616

Arum maculatum agg. 622

Arum maculatum 625

Arum maculatum agg. 622

Asplenium ruta-muraria 674

Asplenium ruta-muraria 674

Asplenium ruta-muraria subsp. ruta-muraria 676

Aster lanceolatus agg. 15106

Aster lanceolatus 693

Aster parviflorus 15085

Atriplex prostrata agg. 20706

Atriplex calotheca 756

Atriplex hastata 20693

Atriplex hastata agg. 755

Atriplex latifolia 760

Atriplex prostrata 762

Atriplex sagittata 754

Atriplex nitens 20702

Aurinia saxatilis 777

Alyssum saxatile 354

Betonica officinalis 826

Betonica officinalis 826

Stachys officinalis 24165

Betula alba agg. 99014

Betula pendula 829

Betula verrucosa 20786

Betula pubescens 830

Betula pubescens 830

Betula pubescens subsp. carpatica 6472

Betula pubescens subsp. pubescens 6471

Bidens frondosa 834

Bidens frondosa 834

Bidens melanocarpa 20788

Bidens tripartita 836

Bidens tripartita 836

Bidens tripartita subsp. tripartita 838

Biscutella laevigata 844

Biscutella laevigata 844

Biscutella laevigata subsp. kerneri 6652

Bistorta officinalis 27744

Bistorta officinalis 27744

Persicaria bistorta 23029

Polygonum bistorta 4420

Bistorta vivipara 20801

Bistorta vivipara 20801

Persicaria vivipara 23045

Polygonum viviparum 4437

Bolboschoenus maritimus 852

Bolboschoenus maritimus 852

Schoenoplectus maritimus 23820

Bothriochloa ischaemum 854

Andropogon ischaemum 20417

Bothriochloa ischaemum 854

Dichanthium ischaemum 21538

Brachypodium pinnatum agg. 862

Brachypodium pinnatum 863

Brachypodium pinnatum agg. 862

Brachypodium rupestre 864

Bromus arvensis 883

Bromus arvensis 883

Bromus arvensis subsp. arvensis 7191

Bromus hordeaceus agg. 895

Bromus hordeaceus 896

Bromus hordeaceus agg. 895

Bromus hordeaceus subsp. hordeaceus 897

Bromus mollis 901

Bromus racemosus agg. 908

Bromus commutatus 909

Bromus racemosus 910

Bromus ramosus agg. 911

Bromus benekenii 912

Bromus ramosus 913

Bromus ramosus agg. 911

Callitriche palustris agg. 999

Callitriche obtusangula 1003

Callitriche palustris 1004

Callitriche palustris agg. 999

Callitriche stagnalis 1006

Campanula rotundifolia agg. 1057

Campanula rotundifolia 1072

Campanula rotundifolia agg. 1057

Campanula scheuchzeri 1073

Cardamine bulbifera 20944

Cardamine bulbifera 20944

Dentaria bulbifera 1896

Cardamine heptaphylla 20951

Dentaria heptaphyllos 91015

Cardamine pratensis agg. 1105

Cardamine dentata 20945

Cardamine palustris 1109

Cardamine pratensis 15133

Cardamine pratensis agg. 1105

Cardaminopsis arenosa 1114

Arabis arenosa 20509

Cardaminopsis arenosa 1114

Cardaminopsis halleri 1116

Arabis halleri 20518

Cardaminopsis halleri 1116

Cardaminopsis petraea 1117

Arabis hispida 20526

Carduus nutans agg. 1140

Carduus nutans 1143

Carduus nutans agg. 1140

Carex acuta agg. 26775

Carex acuta 20989

Carex acuta subsp. tricostata 6685

Carex gracilis 1230

Carex arenaria agg. 1159

Carex arenaria 1160

Carex ligerica 1161

Carex pseudobrizoides 1162

Carex atrata agg. 1164

Carex atrata 14018

Carex atrata agg. 1164

Carex elata 1204

Carex elata 1204

Carex elata subsp. elata 1205

Carex flacca 1216

Carex flacca 1216

Carex glauca 21026

Carex flava agg. 1219

Carex demissa 20011

Carex flava 1220

Carex flava agg. 1219

Carex flava var. oederi 25010

Carex lepidocarpa 1222

Carex oederi 1223

Carex serotina 21070

Carex tumidicarpa 1225

Carex viridula 6693

Carex muricata agg. 1249

Carex divulsa 1250

Carex leersiana 1251

Carex muricata 6687

Carex muricata agg. 1249

Carex muricata subsp. muricata 6689

Carex pairae 1252

Carex spicata 1253

Carex nigra agg. 1254

Carex fusca 21024

Carex nigra 1256

Carex nigra agg. 1254

Carex ornithopoda agg. 1262

Carex ornithopoda 1263

Carex ornithopoda agg. 1262

Carex ovalis 21055

Carex leporina 1240

Carex ovalis 21055

Carex vulpina agg. 1301

Carex otrubae 1302

Carex vulpina 1303

Carex vulpina agg. 1301

Carex × elytroides 10122

Carex × elytroides 10122

Carex acuta × nigra 90526

Carex × figertii 90596

Carex davalliana × dioica 90549

Carex × xanthocarpa 10137

Carex flava × hostiana 90562

Carlina acaulis 1306

Carlina acaulis 1306

Carlina acaulis subsp. simplex 1308

Carlina vulgaris 1312

Carlina vulgaris 1315

Carlina vulgaris subsp. vulgaris 21094

Castanea sativa 1323

Castanea sativa 1323

Castanea vesca 21097

Caucalis platycarpos 1329

Caucalis lappula 21108

Caucalis platycarpos 1329

Centaurea jacea 1347

Centaurea angustifolia 21122

Centaurea jacea 1347

Centaurea jacea agg. 24981

Centaurea jacea subsp. amara 21132

Centaurea jacea subsp. angustifolia 1348

Centaurea nigra 26577

Centaurea nemoralis 21138

Centaurea nigra 26577

Centaurea nigra subsp. nemoralis 1370

Centaurea scabiosa 1390

Centaurea scabiosa 1390

Centaurea scabiosa subsp. scabiosa 1397

Centaurea stoebe 25004

Centaurea maculosa subsp. rhenana 21134

Centaurea rhenana 21146

Centaurea stoebe 25004

Centaurium erythraea 1406

Centaurium erythraea 1406

Centaurium erythraea subsp. erythraea 1407

Centaurium umbellatum 21156

Centaurium littorale 1409

Centaurium littorale 1409

Centaurium minus 7104

Cerastium arvense 1431

Cerastium arvense 1431

Cerastium arvense subsp. arvense 1432

Cerastium fontanum agg. 1449

Cerastium caespitosum 21178

Cerastium fontanum 1450

Cerastium fontanum agg. 1449

Cerastium fontanum subsp. triviale 21187

Cerastium holosteoides 1451

Cerastium triviale 25269

Cerastium vulgare 21213

Cerastium pumilum agg. 1460

Cerastium glutinosum 1461

Cerastium pallens 21198

Cerastium pumilum 1462

Cerastium pumilum agg. 1460

Ceratocapnos claviculata 21221

Ceratocapnos claviculata 21221

Corydalis claviculata 1670

Cerinthe glabra 1473

Cerinthe alpina 21227

Chaerophyllum hirsutum 26947

Chaerophyllum hirsutum 26947

Chaerophyllum hirsutum agg. 1490

Chaerophyllum villarsii 1493

Chamaecytisus ratisbonensis 1504

Chamaecytisus ratisbonensis 1504

Cytisus ratisbonensis 21484

Chamaecytisus supinus 1506

Chamaecytisus supinus 1506

Cytisus supinus 21486

Chamaespartium sagittale 1509

Chamaespartium sagittale 1509

Genista sagittalis 21992

Genistella sagittalis 2615

Chenopodium album agg. 1514

Chenopodium album 1515

Chenopodium album agg. 1514

Chenopodium strictum subsp. striatiforme 6482

Cirsium acaule 1556

Cirsium acaule 1556

Cirsium acaulon 21327

Cirsium heterophyllum 1569

Cirsium helenioides 21329

Cirsium heterophyllum 1569

Cirsium vulgare 1579

Cirsium lanceolatum 21330

Cirsium vulgare 1579

Cirsium × rigens 26068

Cirsium acaule × oleraceum 90817

Clinopodium vulgare 1593

Calamintha clinopodium 978

Consolida regalis 1627

Consolida regalis 1627

Delphinium consolida 21510

Convolvulus 60816

Convolvulus 60816

Convolvulus arvensis 1632

Conyza canadensis 1638

Conyza canadensis 1638

Erigeron canadensis 21687

Crataegus 61165

Crataegus curvisepala 1697

Crataegus laevigata agg. 1701

Crataegus laevigata 1701

Crataegus lindmanii 1699

Crataegus monogyna 1707

Crataegus monogyna subsp. monogyna 1708

Crataegus monogyna subsp. nordica 1709

Crataegus monogyna var. monogyna 90937

Crataegus oxyacantha 21400

Crataegus rhipidophylla 26677

Crataegus × macrocarpa 1705

Crataegus × media 50021

Crataegus calycina 6733

Crepis bocconi 21408

Crepis pontana 1735

Cruciata laevipes 1766

Cruciata laevipes 1766

Galium cruciata 21963

Cystopteris fragilis agg. 1825

Cystopteris alpina 21473

Cystopteris fragilis 1827

Cytisus nigricans 21483

Cytisus nigricans 21483

Lembotropis nigricans 3333

Cytisus scoparius 1837

Cytisus scoparius 1837

Sarothamnus scoparius 5245

Dactylis glomerata agg. 1842

Dactylis 397

Dactylis aschersoniana 21487

Dactylis glomerata 1843

Dactylis glomerata agg. 1842

Dactylis glomerata subsp. glomerata 13464

Dactylis polygama 1846

Dactylorhiza maculata agg. 1852

Dactylorhiza fuchsii 1853

Dactylorhiza fuchsii × maculata 90986

Dactylorhiza maculata 1857

Dactylorhiza maculata agg. 1852

Dactylorhiza majalis agg. 1861

Dactylorhiza majalis 1862

Dactylorhiza majalis agg. 1861

Dactylorhiza majalis subsp. majalis 1866

Dactylorhiza traunsteineri 1871

Dactylorhiza × carnea 13760

Dactylorhiza maculata × incarnata 90995

Danthonia decumbens 1874

Danthonia decumbens 1874

Danthonia decumbens subsp. decumbens 1876

Sieglingia decumbens 24019

Daucus carota 1886

Daucus 61271

Daucus carota 1886

Daucus carota subsp. carota 6753

Deschampsia cespitosa agg. 1903

Deschampsia cespitosa 1904

Deschampsia cespitosa agg. 1903

Deschampsia flexuosa 20725

Aira flexuosa 20226

Avenella flexuosa 783

Deschampsia flexuosa 20725

Dianthus gratianopolitanus 1934

Dianthus caesius 21522

Dianthus gratianopolitanus 1934

Dianthus superbus 1949

Dianthus superbus 1949

Dianthus superbus subsp. superbus 1952

Digitalis grandiflora 1960

Digitalis ambigua 21540

Dipsacus fullonum 1980

Dipsacus fullonum 1980

Dipsacus sylvestris 20021

Draba aizoides agg. 2000

Draba aizoides 2001

Draba aizoides agg. 2000

Drosera longifolia 21588

Drosera anglica 2025

Drosera longifolia 21588

Dryopteris carthusiana agg. 2030

Dryopteris × deweveri 50029

Dryopteris carthusiana 2032

Dryopteris carthusiana × dilatata 91118

Dryopteris carthusiana agg. 2030

Dryopteris dilatata 2033

Dryopteris expansa 2031

Dryopteris spinulosa 21604

Dryopteris filix-mas agg. s. l. 94728

Dryopteris affinis 2038

Dryopteris filix-mas 2037

Dryopteris filix-mas agg. 2035

Eleocharis palustris agg. 2087

Eleocharis palustris 2091

Eleocharis palustris agg. 2087

Eleocharis uniglumis 2094

Scirpus palustris 23856

Elymus arenosus 27779

Elymus repens subsp. arenosus 6543

Elymus athericus 27782

Elymus pungens 27905

Elymus caninus 20145

Agropyron caninum 103

Elymus caninus 20145

Roegneria canina 23598

Elymus repens 27778

Agropyron repens 27914

Agropyron repens subsp. caesium 6541

Elymus repens 27778

Elymus repens subsp. repens 27781

Elytrigia repens 21639

Triticum repens 24393

Empetrum nigrum agg. 2103

Empetrum nigrum 2105

Empetrum nigrum agg. 2103

Epilobium angustifolium 2113

Chamaenerion angustifolium 21237

Epilobium angustifolium 2113

Epilobium ciliatum 21642

Epilobium adenocaulon 2109

Epilobium ciliatum 21642

Epilobium tetragonum 2126

Epilobium tetragonum 2126

Epilobium tetragonum subsp. lamyi 2127

Epilobium tetragonum subsp. tetragonum 2128

Epipactis atrorubens 2130

Epipactis atropurpurea 21653

Epipactis atrorubens 2130

Epipactis rubiginosa 21664

Epipactis helleborine agg. 2131

Epipactis helleborine 2134

Epipactis latifolia 21660

Equisetum fluviatile 2143

Equisetum fluviatile 2143

Equisetum limosum 21674

Equisetum × mildeanum 91305

Equisetum pratense × sylvaticum 91302

Erica carnea 21680

Erica carnea 21680

Erica herbacea 2163

Erigeron acris 2167

Erigeron acris 2167

Erigeron acris subsp. acris 2168

Erigeron annuus 2178

Erigeron annuus 2178

Erigeron strigosus 21695

Erigeron glabratus 21689

Erigeron glabratus 21689

Erigeron polymorphus 2186

Erodium cicutarium agg. 2197

Erodium cicutarium 2199

Erodium cicutarium agg. 2197

Erophila verna 12356

Draba verna 21586

Erophila verna 12356

Erophila verna agg. 2205

Erophila verna subsp. verna 21715

Erysimum maschallianum 2229

Erysimum durum 21726

Erysimum odoratum 2234

Erysimum erysimoides 21727

Erysimum odoratum 2234

Euphorbia verrucosa 2309

Euphorbia brittingeri 21740

Euphorbia verrucosa 2309

Euphrasia officinalis 13816

Euphrasia officinalis 13816

Euphrasia officinalis subsp. rostkoviana 26254

Euphrasia rostkoviana 2332

Euphrasia rostkoviana agg. 2329

Fallopia convolvulus 2359

Fallopia convolvulus 2359

Polygonum convolvulus 23228

Festuca brevipila 26591

Festuca brevipila 26591

Festuca duriuscula 21820

Festuca trachyphylla 2406

Festuca filiformis 21824

Festuca capillata 21813

Festuca filiformis 21824

Festuca tenuifolia 2405

Festuca ovina agg. 2385

Festuca guestfalica et ovina 91404

Festuca guestfalica 6483

Festuca lemanii 94730

Festuca ovina 2396

Festuca ovina s. l. 91415

Festuca ovina subsp. ovina 91418

Festuca ovina var. firmula 21845

Festuca ovina agg. 2385

Festuca ovina subsp. glauca 10352

Festuca pallens 2397

Festuca cinerea 2387

Festuca glauca 2392

Festuca glaucina 91400

Festuca pallens 2397

Festuca pallens * glaucina 91420

Festuca pallens subsp. pallens 6792

Festuca quadriflora 21852

Festuca pumila 2416

Festuca quadriflora 21852

Festuca rubra agg. 2417

Festuca heteromalla 7346

Festuca nigrescens 2420

Festuca nigrescens subsp. nigrescens 91414

Festuca rubra 2421

Festuca rubra agg. 2417

Festuca rubra subsp. commutata 21856

Festuca rubra subsp. fallax 13510

Festuca rubra subsp. rubra 2425

Festuca rubra var. genuina 21863

Festuca trichophylla 2426

Festuca rupicola 2402

Festuca rupicola 2402

Festuca sulcata 27166

Festuca valesiaca subsp. sulcata 10354

Festuca valesiaca 13712

Festuca pulchra 13523

Festuca valesiaca 13712

Festuca violacea agg. 2441

Festuca norica 2443

Festuca violacea agg. 2441

Filipendula vulgaris 2462

Filipendula hexapetala 21900

Filipendula vulgaris 2462

Frangula alnus 2472

Frangula alnus 2472

Rhamnus frangula 23535

Fumana procumbens 2486

Fumana procumbens 2486

Fumana vulgaris 21921

Galeopsis ladanum agg. 2520

Galeopsis angustifolia 2521

Galeopsis ladanum 2522

Galeopsis tetrahit agg. 2526

Galeopsis bifida 2527

Galeopsis tetrahit 2528

Galeopsis tetrahit agg. 2526

Galium aparine agg. 2532

Galium aparine 2533

Galium aparine agg. 2532

Galium spurium 2534

Galium glaucum 2542

Asperula glauca 20637

Galium glaucum 2542

Galium mollugo agg. 2548

Galium album 2549

Galium album subsp. album 2550

Galium mollugo 2555

Galium mollugo agg. 2548

Galium palustre 2564

Galium elongatum 2563

Galium palustre 2564

Galium palustre agg. 2561

Galium palustre subsp. elongatum 21974

Galium palustre subsp. palustre 21975

Galium pusillum agg. 2569

Galium anisophyllon 2570

Galium pumilum 2572

Galium pusillum agg. 2569

Galium valdepilosum 2577

Galium rotundifolium 2579

Galium scabrum 21982

Galium saxatile 2585

Galium harcynicum 2543

Galium saxatile 2585

Galium verum agg. s. l. 94733

Galium × pomeranicum 2599

Galium verum 2600

Galium verum agg. 2598

Galium verum subsp. verum 24929

Galium wirtgenii 2601

Gentiana acaulis agg. 91530

Gentiana acaulis 2616

Gentiana clusii 2624

Gentianella ciliata 2656

Gentiana ciliata 22041

Gentianella ciliata 2656

Gentianopsis ciliata 22040

Gentianella germanica agg. 2657

Gentiana germanica 22044

Gentianella aspera 2659

Gentianella germanica 2662

Gentianella germanica agg. 2657

Gentianella germanica subsp. germanica 7352

Gentianella lutescens 2665

Geum × intermedium 50040

Geum rivale × urbanum 91547

Glechoma hederacea agg. 2710

Glechoma hederacea 2711

Glechoma hederacea agg. 2710

Globularia punctata 2718

Globularia bisnagarica 2718

Globularia elongata 22079

Globularia punctata 2718

Globularia vulgaris 26117

Globularia willkommii 22080

Glyceria fluitans agg. 2719

Glyceria declinata 2720

Glyceria fluitans 2721

Glyceria fluitans agg. 2719

Glyceria notata 26260

Helianthemum nummularium 26596

Helianthemum nummularium 26596

Helianthemum nummularium agg. 2774

Helianthemum nummularium subsp. obscurum 22127

Helianthemum nummularium subsp. ovatum 22128

Helianthemum ovatum 2780

Helictotrichon pratense 20728

Avena pratensis 20717

Avenochloa pratensis 788

Avenula pratensis 20732

Helictotrichon pratense 20728

Helictotrichon pubescens 20729

Avena pubescens 20718

Avenochloa pubescens 789

Avenula pubescens 20733

Helictotrichon pubescens 20729

Helictotrichon versicolor 20730

Avenula versicolor 20734

Hepatica nobilis 2815

Anemone hepatica 20422

Hepatica nobilis 2815

Hepatica triloba 10391

Herniaria glabra 2832

Herniaria glabra 2832

Herniaria glabra subsp. glabra 2833

Hieracium angustifolium 25621

Hieracium glaciale 2885

Hieracium caespitosum 2866

Pilosella caespitosa 12173

Hieracium lactucella 2899

Hieracium auricula 2857

Hieracium lactucella 2899

Pilosella lactucella 12147

Hieracium murorum 25659

Hieracium murorum 25659

Hieracium sylvaticum 2942

Hieracium pilosella 2923

Hieracium pilosella 2923

Pilosella officinarum 14992

Hieracium piloselloides 2924

Hieracium piloselloides 2924

Pilosella piloselloides 12226

Hieracium rothianum 25654

Hieracium setigerum 12219

Hieracium subramosum 10562

depr. Hieracium subramosum 94848

Hippocrepis emerus 21379

Coronilla emerus 1655

Hornungia petraea 2993

Hornungia petraea 2993

Hutchinsia petraea 22188

Hypericum maculatum agg. 3027

Hypericum × desetangsii 3029

Hypericum maculatum 20046

Hypericum maculatum agg. 3027

Hypericum maculatum subsp. maculatum 20047

Hypericum perforatum 3036

Hypericum perforatum 3036

Hypericum perforatum subsp. angustifolium 22208

Hypericum tetrapterum 3042

Hypericum quadrangulum 22214

Hypericum tetrapterum 3042

Inula conyzae 22227

Inula conyza 3069

Inula conyzae 22227

Jasione laevis 3117

Jasione perennis 22240

Juncus alpinus 22243

Juncus alpinoarticulatus 91853

Juncus alpinus 22243

Juncus articulatus 3136

Juncus articulatus 3136

Juncus supinus 22278

Juncus bufonius agg. 3140

Juncus bufonius 3141

Juncus bufonius agg. 3140

Juncus compressus agg. 3147

Juncus compressus 3148

Juncus gerardii 3149

Juncus trifidus 26603

Juncus monanthos 3165

Juncus trifidus subsp. monanthos 22281

Juniperus communis 3168

Juniperus communis 3168

Juniperus communis subsp. communis 3170

Kobresia myosuroides 22299

Elyna myosuroides 2102

Kobresia myosuroides 22299

Koeleria pyramidata agg. 3233

Koeleria 61398

Koeleria cristata 22305

Koeleria gracilis 22312

Koeleria macrantha 3235

Koeleria pyramidata 3236

Koeleria pyramidata agg. 3233

Lamium galeobdolon agg. 22337

Galeobdolon argentatum 21942

Galeobdolon luteum 20026

Galeobdolon luteum agg. 91498

Galeobdolon montanum 20027

Lamiastrum galeobdolon 3256

Lamiastrum montanum 3257

Lamium argentatum 24905

Lamium galeobdolon 22338

Lamium galeobdolon agg. 22337

Lamium montanum 22340

Larix decidua 3272

Larix decidua 3272

Larix europaea 10606

Lathyrus linifolius 3301

Lathyrus linifolius 3301

Lathyrus montanus 22390

Ledum palustre 3328

Ledum palustre 3328

Rhododendron tomentosum 28144

Leontodon helveticus 3346

Leontodon helveticus 3346

Leontodon pyrenaicus subsp. helveticus 7365

Leontodon hispidus 3347

Leontodon hastilis 25306

Leontodon hispidus 3347

Leontodon hispidus subsp. hastilis 22413

Leontodon hispidus subsp. hispidus 6837

Leontodon saxatilis 3354

Leontodon saxatilis 3354

Leontodon taraxacoides 22424

Leucanthemum atratum agg. 3375

Leucanthemum atratum agg. 3375

Leucanthemum halleri 3378

Leucanthemum vulgare agg. 3381

Chrysanthemum leucanthemum 21299

Leucanthemum ircutianum 3384

Leucanthemum maximum 3386

Leucanthemum vulgare 3387

Leucanthemum vulgare agg. 3381

Linaria vulgaris agg. 3429

Linaria vulgaris 3433

Linaria vulgaris agg. 3429

Lindernia dubia 3434

Gratiola anagallidea 13832

Linum perenne agg. 3454

Linum austriacum 3455

Linum leonii 3458

Linum perenne 26993

Lithospermum arvense 3475

Buglossoides arvensis subsp. sibthorpiana 20947

Lithospermum arvense 3475

Lotus corniculatus agg. 3505

Lotus corniculatus 3508

Lotus corniculatus agg. 3505

Lotus corniculatus subsp. corniculatus 22509

Lotus corniculatus var. ciliatus 25841

Lotus tenuis 3510

Lotus pedunculatus 22517

Lotus pedunculatus 22517

Lotus uliginosus 3516

Luzula campestris agg. 3530

Luzula campestris 3531

Luzula campestris agg. 3530

Luzula campestris subsp. multiflora 22525

Luzula multiflora 11817

Luzula pallescens 25388

Luzula sudetica 3536

Luzula luzuloides 3541

Luzula albida 3526

Luzula luzuloides 3541

Luzula luzuloides subsp. rubella 14424

Luzula sylvatica 3547

Luzula sylvatica 3547

Luzula sylvatica subsp. sylvatica 3549

Lycopersicon esculentum 3558

Lycopersicon esculentum 3558

Solanum lycopersicum 24088

Lycopus europaeus 3562

Lycopus europaeus 3562

Lycopus europaeus subsp. europaeus 3563

Malus sylvestris agg. 3580

Malus sylvestris 3582

Malus sylvestris agg. 3580

Matricaria 60765

Chamomilla 67025

Matricaria 60765

Matricaria discoidea 3597

Chamomilla suaveolens 21249

Matricaria discoidea 3597

Matricaria recutita 21248

Chamomilla recutita 21247

Matricaria recutita 21248

Medicago sativa agg. 3616

Medicago × varia 3620

Medicago falcata 3617

Medicago sativa 11820

Medicago sativa agg. 3616

Melampyrum pratense 3638

Melampyrum pratense 3638

Melampyrum pratense subsp. pratense 13853

Melica ciliata agg. 3643

Melica ciliata 3644

Melica ciliata var. nebrodensis 13015

Melica nutans agg. 3648

Melica nutans 3650

Melica nutans agg. 3648

Melica picta 3651

Mentha spicata agg. 3676

Mentha longifolia 3677

Mentha suaveolens 3680

Mercurialis perennis agg. 3691

Mercurialis ovata 3692

Mercurialis perennis 3694

Minuartia hybrida 3720

Alsine tenuifolia 25807

Minuartia rubra 22674

Minuartia fastigiata 3715

Minuartia rubra 22674

Minuartia verna 26607

Minuartia verna 26607

Minuartia verna subsp. gerardii 22675

Minuartia verna subsp. hercynica 6499

Molinia caerulea agg. 3756

Molinia 61408

Molinia arundinacea 3757

Molinia caerulea 3758

Monotropa hypopitys agg. 3762

Monotropa hypophegea 3763

Monotropa hypopitys 3764

Monotropa hypopitys agg. 3762

Montia fontana 3765

Montia fontana 3765

Montia fontana subsp. chondrosperma 3767

Mycelis muralis 3781

Lactuca muralis 22329

Mycelis muralis 3781

Myosotis arvensis 3782

Myosotis arvensis 3782

Myosotis arvensis subsp. arvensis 3783

Myosotis discolor 3785

Myosotis discolor 3785

Myosotis versicolor 22757

Myosotis ramosissima 3792

Myosotis hispida 22735

Myosotis ramosissima 3792

Myosotis scorpioides agg. 20051

Myosotis caespitosa 22731

Myosotis laxa 6500

Myosotis laxiflora 3788

Myosotis multiflora 12098

Myosotis nemorosa 3789

Myosotis nemorosa subsp. brevisetacea 22741

Myosotis palustris 22743

Myosotis palustris agg. 3786

Myosotis palustris s. l. 15315

Myosotis scorpioides 3791

Myosotis scorpioides agg. 20051

Myosotis scorpioides subsp. scorpioides 12104

Myosotis stricta 3804

Myosotis micrantha 22740

Myosotis stricta 3804

Myosotis sylvatica agg. 3793

Myosotis alpestris 3794

Myosotis decumbens 3795

Myosotis sylvatica 3802

Nigritella nigra agg. 3851

Nigritella nigra 7167

Nigritella nigra agg. 3851

Odontites luteus 3863

Euphrasia lutea 21768

Odontites luteus 3863

Orthanthella lutea 3863

Odontites vernus agg. 12023

Odontites ruber 13749

Odontites vernus 3866

Odontites vernus agg. 12023

Odontites vulgaris 3867

Onobrychis viciifolia agg. 3906

Onobrychis arenaria 3907

Onobrychis sativa 93791

Onobrychis viciifolia 3912

Ononis spinosa agg. 3920

Ononis procurrens 22831

Ononis repens 3922

Ononis repens subsp. procurrens 3923

Ononis spinosa 3925

Ononis spinosa agg. 3920

Ononis spinosa subsp. maritima 22837

Ophrys holoserica 3955

Ophrys fuciflora 22852

Ophrys holoserica 3955

Ophrys insectifera 3956

Ophrys insectifera 3956

Ophrys muscifera 22859

Oreopteris limbosperma 22909

Dryopteris montana 21597

Lastrea limbosperma 22377

Oreopteris limbosperma 22909

Thelypteris limbosperma 5910

Orthilia secunda 4054

Orthilia secunda 4054

Pyrola secunda 23440

Oxalis stricta 22973

Oxalis fontana 4065

Oxalis stricta 22973

Oxytropis montana agg. 4080

Oxytropis jacquinii 4084

Oxytropis montana agg. 4080

Papaver dubium 10700

Papaver dubium 10700

Papaver dubium subsp. lecoqii 22999

Pedicularis palustris 4158

Pedicularis palustris 4158

Pedicularis palustris subsp. palustris 13828

Persicaria amphibia 23027

Persicaria amphibia 23027

Polygonum amphibium 4413

Persicaria dubia 11734

Persicaria dubia 11734

Persicaria mitis 23041

Polygonum mite 4429

Persicaria hydropiper 23032

Persicaria hydropiper 23032

Polygonum hydropiper 4422

Persicaria lapathifolia 23034

Persicaria lapathifolia 23034

Persicaria lapathifolia subsp. pallida 13950

Polygonum lapathifolium 4423

Persicaria maculosa 23038

Persicaria maculosa 23038

Polygonum persicaria 4435

Persicaria minor 23039

Persicaria minor 23039

Polygonum minus 4428

Petrorhagia prolifera agg. 4185

Petrorhagia prolifera 4187

Tunica prolifera 24402

Petrorhagia saxifraga 4189

Petrorhagia saxifraga 4189

Tunica saxifraga 24403

Phegopteris connectilis 23065

Gymnocarpium phegopteris 12299

Phegopteris connectilis 23065

Thelypteris phegopteris 5912

Phleum alpinum agg. 4215

Phleum alpinum 25110

Phleum alpinum agg. 4215

Phleum phleoides 4222

Phleum boehmeri 23073

Phleum phleoides 4222

Phleum pratense agg. 4223

Phleum bertolonii 4224

Phleum pratense 4225

Phleum pratense agg. 4223

Phleum pratense subsp. pratense 27013

Phragmites australis 4229

Phragmites australis 4229

Phragmites communis 23084

Phyteuma orbiculare 26610

Phyteuma orbiculare 26610

Phyteuma orbiculare subsp. orbiculare 24940

Phyteuma spicatum 4264

Phyteuma spicatum 4264

Phyteuma spicatum subsp. occidentale 14758

Phyteuma spicatum subsp. spicatum 4266

Phyteuma spicatum var. coeruleum 23091

Picea abies 4269

Picea abies 4269

Picea excelsa 23106

Picris hieracioides 4274

Picris hieracioides 4274

Picris hieracioides agg. 4272

Pimpinella major 4277

Pimpinella major 4277

Pimpinella major subsp. major 23108

Pimpinella saxifraga agg. 4279

Pimpinella saxifraga 4282

Pimpinella saxifraga agg. 4279

Plantago lanceolata 4320

Plantago lanceolata 4320

Plantago lanceolata subsp. sphaerostachya 23153

Plantago lanceolata var. sphaerostachya 25683

Plantago major 4321

Plantago intermedia 23150

Plantago major 4321

Plantago major subsp. intermedia 4322

Plantago major subsp. major 4323

Plantago maritima agg. 4325

Plantago alpina 4326

Plantago maritima 27766

Plantago strictissima 23161

Plantago media agg. 4332

Plantago media 4333

Plantago media agg. 4332

Poa annua agg. 4343

Poa annua 4344

Poa annua agg. 4343

Poa supina 4345

Poa pratensis agg. 4366

Poa angustifolia 4367

Poa humilis 7372

Poa pratensis 4368

Poa pratensis agg. 4366

Poa pratensis subsp. angustifolia 23193

Poa trivialis 26611

Poa trivialis 26611

Poa trivialis agg. 4372

Poa trivialis subsp. trivialis 7161

Polygala amara agg. 4390

Polygala amara 4391

Polygala amara agg. 4390

Polygala amara subsp. amarella 23205

Polygala amarella 4394

Polygala vulgaris 4405

Polygala oxyptera 23212

Polygala vulgaris 4405

Polygala vulgaris s. str. 10745

Polygala vulgaris subsp. oxyptera 4406

Polygala vulgaris subsp. vulgaris 4407

Polygonatum odoratum 4410

Polygonatum odoratum 4410

Polygonatum officinale 27134

Polygonum aviculare agg. 4415

Polygonum aviculare 4417

Polygonum aviculare agg. 4415

Populus × canadensis 50080

Populus × canadensis 50080

Populus × euamericana 10760

Potentilla argentea agg. 4490

Potentilla argentea 4491

Potentilla argentea agg. 4490

Potentilla erecta 4511

Potentilla erecta 4511

Potentilla tormentilla 23354

Potentilla heptaphylla agg. 4515

Potentilla heptaphylla 4517

Potentilla rubens 23350

Potentilla incana 25757

Potentilla arenaria 4539

Potentilla cinerea 25853

Potentilla cinerea subsp. incana 15377

Potentilla incana 25757

Potentilla × subarenaria 50084

Potentilla palustris 4528

Comarum palustre 21352

Potentilla palustris 4528

Potentilla verna agg. 4538

Potentilla neumanniana 4541

Potentilla pusilla 4542

Potentilla tabernaemontani 20053

Potentilla verna 23355

Potentilla verna agg. 4538

Potentilla verna subsp. vulgaris 28077

Primula veris 4570

Primula officinalis 23378

Primula veris 4570

Primula veris subsp. veris 4575

Primula × digenea 92537

Primula elatior × vulgaris 92534

Primula × media 50085

Primula elatior × veris 92533

Pritzelago alpina 26680

Hutchinsia alpina 3000

Pritzelago alpina 26680

Prunus avium 4582

Cerasus avium 21216

Prunus avium 4582

Prunus avium subsp. avium 24942

Prunus cerasus agg. 4585

Cerasus vulgaris 21220

Prunus cerasus 4586

Prunus mahaleb 4592

Cerasus mahaleb 21219

Prunus mahaleb 4592

Prunus padus 4593

Padus avium 22980

Prunus padus 4593

Prunus spinosa agg. 4597

Prunus × fruticans 4598

Prunus spinosa 4599

Prunus spinosa subsp. fruticans 23396

Pseudolysimachion longifolium 23400

Pseudolysimachion longifolium 23400

Veronica longifolia 6261

Pseudolysimachion spicatum 23404

Pseudolysimachion spicatum 23404

Veronica spicata 6286

Pseudorchis albida 4601

Leucorchis albida 3390

Pseudorchis albida 4601

Pulmonaria officinalis agg. 4636

Pulmonaria obscura 4637

Pulmonaria officinalis 4638

Pulmonaria officinalis agg. 4636

Pulsatilla alpina 15385

Pulsatilla alpina agg. 4642

Pulsatilla vulgaris 26617

Anemone pulsatilla 20428

Anemone pulsatilla subsp. grandis 20429

Pulsatilla vulgaris 26617

Pulsatilla vulgaris agg. 4656

Pulsatilla vulgaris subsp. grandis 23437

Pyrus communis agg. 4669

Pyrus communis 6936

Pyrus communis agg. 4669

Pyrus communis subsp. pyraster 23449

Pyrus pyraster 4671

Quercus petraea agg. 4678

Quercus petraea 4680

Quercus sessiliflora 23468

Quercus robur 4685

Quercus pedunculata 23466

Quercus robur 4685

Ranunculus acris agg. 4689

Ranunculus acris 4690

Ranunculus acris agg. 4689

Ranunculus aquatilis agg. 4697

Ranunculus aquatilis 4698

Ranunculus aquatilis agg. 4697

Ranunculus peltatus 12030

Ranunculus auricomus agg. 4709

Ranunculus auricomus 11978

Ranunculus auricomus agg. 4709

Ranunculus auricomus s. l. 4710

Ranunculus biformis 27241

Ranunculus kochii 10821

Ranunculus rectus 6956

Ranunculus bulbosus 4717

Ranunculus bulbosus 4717

Ranunculus bulbosus subsp. bulbosus 12015

Ranunculus ficaria 4721

Ficaria verna 21893

Ranunculus ficaria 4721

Ranunculus ficaria subsp. bulbilifer 4722

Ranunculus flammula agg. 4726

Ranunculus flammula 4727

Ranunculus flammula agg. 4726

Ranunculus reptans 4728

Ranunculus montanus agg. 4743

Ranunculus breyninus 23477

Ranunculus montanus 4747

Ranunculus montanus agg. 4743

Ranunculus polyanthemos agg. 4758

Ranunculus nemorosus 4759

Ranunculus nemorosus subsp. polyanthemophyllus 23501

Ranunculus polyanthemoides 4760

Ranunculus polyanthemophyllus 4761

Ranunculus polyanthemos 4762

Ranunculus polyanthemos agg. 4758

Raphanus raphanistrum agg. 4778

Raphanus raphanistrum 4780

Raphanus sativus 4781

Rhinanthus angustifolius 23544

Rhinanthus angustifolius 23544

Rhinanthus angustifolius subsp. angustifolius 23545

Rhinanthus angustifolius subsp. grandiflorus 6569

Rhinanthus serotinus 4832

Rhinanthus aristatus agg. 4817

Rhinanthus aristatus 23551

Rhinanthus glacialis 4819

Rhinanthus minor 4828

Alectorolophus minor 20304

Rhinanthus minor 4828

Ribes rubrum agg. 4850

Ribes rubrum 4851

Ribes rubrum agg. 4850

Rosa canina agg. s. l. 94740

Rosa canina 26665

Rosa canina agg. 4872

Rosa canina agg. s. l. 94740

Rosa corymbifera 26666

Rosa obtusifolia 4897

Rosa rubiginosa agg. s. l. 92752

Rosa elliptica 4887

Rosa micrantha 4893

Rosa rubiginosa 4902

Rosa rubiginosa agg. s. l. 92752

Rosa spinosissima 23649

Rosa pimpinellifolia 4899

Rosa tomentosa agg. s. l. 92754

Rosa tomentosa 4912

Rosa tomentosa agg. 4907

Rosa villosa agg. 4908

Rubus canescens 4952

Rubus canescens 4952

Rubus tomentosus 25709

Rubus grabowskii 7078

Rubus thyrsanthus 5056

Rubus latiarcuatus 24749

Rubus vulgaris var. mollis 11374

Rubus montanus 24778

Rubus candicans 4951

Rubus montanus 24778

Rubus plicatus 5024

Rubus affinis 4931

Rubus plicatus 5024

Rubus sect. Rubus 11351

Rubus fruticosus s. l. 11886

Rubus sect. Rubus 11351

Rumex acetosa agg. 92799

Rumex acetosa 5073

Rumex thyrsiflorus 5108

Rumex acetosella 26619

Rumex acetosella 26619

Rumex acetosella agg. 5074

Rumex acetosella subsp. acetosella 26618

Rumex acetosella subsp. tenuifolius 27201

Rumex tenuifolius 5077

Rumex arifolius 20080

Rumex alpestris 5078

Rumex arifolius 20080

Rumex pseudoalpinus 23661

Rumex alpinus 5079

Rumex × pratensis 50156

Rumex × pratensis 50156

Rumex crispus × obtusifolius 92814

Salix fragilis agg. 5167

Salix × rubens 5169

Salix fragilis 5168

Salix myrsinifolia 5185

Salix myrsinifolia 5185

Salix nigricans 23730

Salix repens agg. 27193

Salix repens 27194

Salix repens agg. 27193

Salix repens s. l. 5191

Salix repens subsp. repens 5193

Salix retusa agg. 5196

Salix retusa 5198

Salix retusa agg. 5196

Salix serpillifolia 5199

Salix × multinervis 27195

Salix × multinervis 27195

Salix aurita × cinerea 92857

Salix × smithiana 27196

Salix × smithiana 27196

Salix caprea × viminalis 92873

Salix × wimmeriana 50108

Salix caprea × purpurea 92872

Salsola kali 5208

Salsola kali subsp. tragus 5211

Salsola tragus 23749

Sanguisorba minor 5231

Sanguisorba minor 5231

Sanguisorba minor subsp. minor 5232

Scabiosa columbaria agg. 5337

Scabiosa columbaria 5338

Scabiosa columbaria agg. 5337

Scabiosa lucida 5342

Scleranthus annuus agg. 5376

Scleranthus annuus 5377

Scleranthus annuus agg. 5376

Scleranthus polycarpos 5378

Scleranthus × intermedius 92980

Scleranthus annuus × perennis 92978

Securigera varia 21382

Coronilla varia 1662

Securigera varia 21382

Sedum rupestre agg. 5429

Sedum reflexum 5433

Sedum rupestre 23907

Sedum rupestre subsp. reflexum 23910

Sedum sexangulare 5437

Sedum boloniense 23894

Sedum mite 23901

Sedum sexangulare 5437

Sedum telephium agg. 5440

Hylotelephium maximum 7126

Sedum maximum 5441

Sedum telephium 27746

Sedum telephium agg. 5440

Sedum telephium subsp. maximum 23912

Senecio alpinus 5467

Senecio alpinus 5467

Senecio cordatus 23945

Senecio aquaticus agg. 5468

Senecio aquaticus 5469

Senecio aquaticus agg. 5468

Senecio erraticus 5470

Senecio incanus 5485

Senecio carniolicus 23942

Senecio jacobaea 5494

Jacobaea vulgaris 25789

Senecio jacobaea 5494

Senecio nemorensis agg. 5496

Senecio fuchsii 5498

Senecio hercynicus 5499

Senecio nemorensis 94860

Senecio nemorensis agg. 5496

Senecio ovatus 23947

Senecio ovatus subsp. alpestris 7006

Serratula tinctoria 27047

Serratula tinctoria 27047

Serratula tinctoria agg. 5524

Seseli libanotis 5540

Libanotis montana 22460

Libanotis sibirica 22463

Seseli libanotis 5540

Sesleria varia agg. 5558

Sesleria albicans 23996

Sesleria albicans subsp. albicans 27051

Sesleria albicans var. albicans 15414

Sesleria caerulea 25028

Sesleria caerulea subsp. calcaria 13441

Sesleria varia 24991

Setaria pumila 24008

Setaria glauca 5563

Setaria pumila 24008

Silene 60691

Lychnis 60681

Silene 60691

Silene acaulis agg. 5578

Silene acaulis 5579

Silene acaulis agg. 5578

Silene coronaria 11625

Lychnis coronaria 3551

Silene flos-cuculi 24031

Lychnis flos-cuculi 3552

Silene flos-cuculi 24031

Silene latifolia 7012

Melandrium album 22627

Silene alba 5581

Silene latifolia 7012

Silene latifolia subsp. alba 22546

Silene pratensis 20058

Silene noctiflora 5603

Melandrium noctiflorum 22630

Silene noctiflora 5603

Silene nutans agg. 5604

Silene nutans 5606

Silene nutans agg. 5604

Silene viscaria 11627

Lychnis viscaria 3554

Silene viscaria 11627

Viscaria vulgaris 24569

Silene vulgaris 5629

Silene cucubalus 24029

Silene inflata 25277

Silene vulgaris 5629

Silene vulgaris agg. 20067

Silene vulgaris subsp. vulgaris 5634

Silene vulgaris var. humilis 24046

Soldanella alpicola 24100

Soldanella pusilla s. l. 5678

Solidago virgaurea 5682

Solidago virgaurea 5682

Solidago virgaurea subsp. virgaurea 5684

Sonchus asper 5690

Sonchus asper 5690

Sonchus asper subsp. asper 5691

Sorbus aria agg. 5696

Sorbus aria 5697

Sorbus aria agg. 5696

Sorbus aucuparia 5700

Sorbus aucuparia 5700

Sorbus aucuparia subsp. aucuparia 5701

Spergula pentandra agg. 5730

Spergula morisonii 5731

Spergula vernalis 24154

Spergularia media 5734

Spergularia maritima 24145

Spergularia media 5734

Stellaria alsine 5769

Stellaria alsine 5769

Stellaria uliginosa 5782

Stellaria aquatica 24917

Myosoton aquaticum 3805

Stellaria aquatica 24917

Stellaria media agg. 5774

Stellaria media 5775

Stellaria media agg. 5774

Stellaria pallida 5777

Stellaria nemorum 5778

Stellaria nemorum 5778

Stellaria nemorum subsp. nemorum 5780

Stellaria palustris 11573

Stellaria glauca 24184

Stellaria palustris 11573

Stipa calamagrostis 24197

Achnatherum calamagrostis 47

Stipa pennata agg. 5787

Stipa joannis 5797

Stipa pennata 24203

Stipa pennata var. mediterranea 13411

Stipa pulcherrima 13746

Symphytum officinale 26629

Symphytum officinale 26629

Symphytum officinale agg. 5823

Tanacetum corymbosum agg. 5841

Chrysanthemum corymbosum 21294

Tanacetum corymbosum 5843

Tanacetum parthenium 5845

Chrysanthemum parthenium 21315

Tanacetum parthenium 5845

Tanacetum vulgare 5846

Chrysanthemum vulgare 21314

Tanacetum vulgare 5846

Taraxacum sect. Alpina et Hamata et Ruderalia 93219

Taraxacum sect. Alpina 7424

Taraxacum officinale agg. 7442

Taraxacum sect. Alpina et Hamata et Ruderalia 93219

Taraxacum sect. Ruderalia 7434

Taraxacum officinale 14513

Taraxacum sect. Erythrosperma 7428

Taraxacum erythrospermum 14460

Taraxacum laevigatum 14457

Taraxacum levigatum agg. 14458

Taraxacum sect. Erythrosperma 7428

Taraxacum sect. Palustria 7433

Taraxacum palustre 25514

Taraxacum sect. Palustria 7433

Tephroseris helenitis 23949

Senecio helenitis 5482

Tephroseris helenitis 23949

Tephroseris integrifolia 23953

Senecio integrifolius 5492

Tephroseris integrifolia 23953

Teucrium scorodonia 5889

Teucrium scorodonia 5889

Teucrium scorodonia subsp. scorodonia 5891

Thalictrum minus agg. 5896

Thalictrum minus 5898

Thalictrum minus agg. 5896

Thalictrum minus subsp. saxatile 5902

Thlaspi caerulescens 5928

Noccaea caerulescens 22785

Thlaspi cepaeifolium 25000

Thlaspi rotundifolium 5940

Thlaspi perfoliatum 5938

Microthlaspi perfoliatum 22667

Thlaspi perfoliatum 5938

Thymus praecox agg. 5955

Thymus praecox 5957

Thymus praecox agg. 5955

Thymus praecox subsp. polytrichus 5959

Thymus praecox subsp. praecox 5960

Thymus serpyllum subsp. praecox 13239

Thymus pulegioides agg. 5964

Thymus pulegioides 5965

Thymus pulegioides agg. 5964

Thymus pulegioides subsp. pulegioides 7053

Thymus serpyllum subsp. chamaedrys 13255

Thymus serpyllum agg. 6456

Thymus angustifolius 24265

Thymus serpyllum 5966

Thymus serpyllum agg. 6456

Tragopogon pratensis 7141

Tragopogon minor 5999

Tragopogon orientalis 6000

Tragopogon pratensis 7141

Tragopogon pratensis agg. 5998

Tragopogon pratensis subsp. orientalis 24325

Tragopogon pratensis subsp. pratensis 7100

Trichophorum cespitosum 27072

Trichophorum cespitosum 27072

Trichophorum cespitosum subsp. germanicum 24330

Trifolium dubium agg. 6028

Trifolium dubium 6029

Trifolium dubium agg. 6028

Trifolium minus 24352

Trifolium pratense 6057

Trifolium pratense 6057

Trifolium pratense subsp. pratense 6061

Tripleurospermum maritimum agg. 6096

Matricaria inodora 22000

Matricaria maritima 22580

Matricaria perforata 22003

Tripleurospermum inodorum 20061

Tripleurospermum maritimum 6098

Tripleurospermum perforatum 6097

Ulmus glabra 6137

Ulmus glabra 6137

Ulmus montana 24424

Ulmus scabra 24425

Ulmus minor agg. 6139

Ulmus campestris 24420

Ulmus minor 6140

Utricularia minor agg. 6152

Utricularia minor 6154

Utricularia minor agg. 6152

Vaccinium oxycoccos agg. 6161

Oxycoccus palustris 4070

Vaccinium oxycoccos 6163

Vaccinium oxycoccos agg. 6161

Vaccinium uliginosum 6166

Vaccinium uliginosum 6166

Vaccinium uliginosum agg. 6164

Valeriana officinalis agg. 6177

Valeriana officinalis 6178

Valeriana officinalis agg. 6177

Valeriana pratensis 6179

Valeriana procurrens 6180

Valeriana wallrothii 6183

Veronica agrestis agg. 93432

Veronica agrestis 6226

Veronica persica 6271

Veronica polita 6272

Veronica anagallis-aquatica agg. 6231

Veronica anagallis-aquatica 6232

Veronica anagalloides 6233

Veronica catenata 6234

Veronica austriaca agg. 6237

Veronica austriaca 13751

Veronica austriaca subsp. dentata 24471

Veronica austriaca subsp. teucrium 25019

Veronica prostrata 13869

Veronica teucrium 6243

Veronica chamaedrys 13752

Veronica chamaedrys 13752

Veronica chamaedrys agg. 6248

Veronica hederifolia 26766

Veronica hederifolia 26766

Veronica hederifolia agg. 6257

Veronica hederifolia subsp. hederifolia 7101

Veronica sublobata 6259

Veronica verna agg. 6292

Veronica dillenii 6293

Veronica verna 6294

Veronica verna agg. 6292

Vicia cracca agg. 6301

Vicia cracca 6302

Vicia cracca agg. 6301

Vicia tenuifolia 6306

Vicia sativa agg. 6328

Vicia angustifolia 6329

Vicia sativa 6334

Vicia sativa agg. 6328

Vicia sativa subsp. nigra 24521

Vicia sativa var. nigra 24518

Vicia tetrasperma agg. 6338

Vicia tenuissima 6340

Vicia tetrasperma 6341

Vicia tetrasperma agg. 6338

Vincetoxicum hirundinaria agg. 6349

Cynanchum vincetoxicum 21465

Vincetoxicum hirundinaria 6351

Vincetoxicum officinale 24536

Viola canina agg. 24994

Viola canina 6367

Viola canina subsp. canina 6368

Viola persicifolia 6386

Viola persicifolia 6386

Viola stagnina 6394

Viola silvatica agg. 94746

Viola × bavarica 7420

Viola reichenbachiana 6390

Viola reichenbachiana × riviniana 93487

Viola riviniana 6391

Viola silvatica 24559

Viola tricolor agg. 6397

Viola arvensis 6398

Viola arvensis × tricolor 93457

Viola arvensis subsp. arvensis 7208

Viola tricolor 6402

Viola tricolor subsp. tricolor 6405

Viola tricolor var. arvensis 12796

x Festulolium krasanii 93548

Festuca arundinacea × Lolium multiflorum 91392
