# Supplementary material for: ReSurveyGermany: Vegetation-plot time-series over the past hundred years in Germany
Source: Sci Data. 2022 Oct 19;9:631. doi: 10.1038/s41597-022-01688-6 (PMC9581966; doi:10.1038/s41597-022-01688-6)
Supplement: Supplementary file 3 — Supplementary Table S3 [file 41597_2022_1688_MOESM3_ESM.docx]

Supplementary Table S3: List of all taxon names that were manually harmonised within projects, in addition to the harmonisation across all projects, as shown in Supplementary Table S2. PROJECT_ID and Project_Name refer to the project in Supplementary Table S1, RS_PLOT is the plot resurvey ID, which identifies the groups of plots compared in time, RELEVE_NR is the plot observation ID in the Turboveg 2 database (Table 2). Taxon_name_old is the name given by the original author, while Taxon_name_new_1 and Taxon_name_new_2 refer to newly assigned taxon names. In case of two new names, i.e. the original taxon concept represented by the name was split into two concepts, the cover values of the old taxon were equally divided among the two new taxa.

| PROJECT_ID | Project_Name | RS_Plot | RELEVE_NR | Taxon_name_old | Taxon_name_new_1 | Taxon_name_new_2 |
| --- | --- | --- | --- | --- | --- | --- |
| 6 | Bode (2005) | HEIDE | 331 | Cuscuta | Cuscuta epithymum |  |
| 6 | Bode (2005) | HEIDE | 270 | Fagus | Fagus sylvatica |  |
| 6 | Bode (2005) | HEIDE | 271 | Fagus | Fagus sylvatica |  |
| 23 | Jandt & Leonhardt (unpubl.) | 32 | 138 | Tragopogon | Tragopogon pratensis |  |
| 23 | Jandt & Leonhardt (unpubl.) | 61 | 186 | Tortula | Tortula ruralis agg. |  |
| 23 | Jandt & Leonhardt (unpubl.) | 63 | 190 | Pulsatilla | Pulsatilla pratensis |  |
| 23 | Jandt & Leonhardt (unpubl.) | 56 | 182 | Anthericum | Anthericum ramosum |  |
| 23 | Jandt & Leonhardt (unpubl.) | 80 | 214 | Epipactis | Epipactis atrorubens |  |
| 23 | Jandt & Leonhardt (unpubl.) | 123 | 6 | Trifolium | Trifolium repens |  |
| 23 | Jandt & Leonhardt (unpubl.) | 138 | 36 | Epipactis | Epipactis atrorubens |  |
| 23 | Jandt & Leonhardt (unpubl.) | 146 | 38 | Achillea | Achillea millefolium agg. |  |
| 23 | Jandt & Leonhardt (unpubl.) | 146 | 38 | Tragopogon | Tragopogon pratensis |  |
| 23 | Jandt & Leonhardt (unpubl.) | 205 | 118 | Tragopogon | Tragopogon pratensis |  |
| 23 | Jandt & Leonhardt (unpubl.) | 246 | 120 | Epipactis | Epipactis atrorubens |  |
| 23 | Jandt & Leonhardt (unpubl.) | 251 | 128 | Epipactis | Epipactis atrorubens |  |
| 23 | Jandt & Leonhardt (unpubl.) | 367 | 166 | Tragopogon | Tragopogon pratensis |  |
| 23 | Jandt & Leonhardt (unpubl.) | 373 | 176 | Tragopogon | Tragopogon pratensis |  |
| 23 | Hagen (1996) | G04 | 180 | Festuca | Festuca rupicola |  |
| 23 | Hagen (1996) | G09 | 171 | Anemone | Pulsatilla vulgaris |  |
| 23 | Hagen (1996) | G15 | 183 | Anemone | Pulsatilla vulgaris |  |
| 23 | Hagen (1996) | G23 | 290 | Epipactis | Epipactis atrorubens |  |
| 16 | Hagen (1996) | Z20 | 377 | Epipactis | Epipactis atrorubens |  |
| 16 | Heinrich, Marstaller & Voigt (2012) | A_CF5 | 857 | Orchis | Orchis militaris |  |
| 16 | Heinrich, Marstaller & Voigt (2012) | CAT_16A | 2014 | Orchis | Orchis x hybrida |  |
| 16 | Heinrich, Marstaller & Voigt (2012) | CAT_14C | 2004 | Orchis | Orchis militaris |  |
| 16 | Heinrich, Marstaller & Voigt (2012) | CAT_16B | 2015 | Orchis | Orchis militaris |  |
| 43 | Heinrich, Marstaller & Voigt (2012) | CAT_18A | 2026 | Orchis | Orchis militaris |  |
| 43 | Heinrich, Marstaller & Voigt (2012) | CAT_18D | 2029 | Orchis | Orchis purpurea |  |
| 43 | Heinrich, Marstaller & Voigt (2012) | CAT_18E | 2030 | Orchis | Orchis purpurea |  |
| 43 | Heinrich, Marstaller & Voigt (2012) | CAT_19A | 2032 | Orchis | Orchis purpurea |  |
| 43 | Heinrich, Marstaller & Voigt (2012) | CAT_19B | 2033 | Orchis | Orchis militaris |  |
| 43 | Heinrich, Marstaller & Voigt (2012) | CAT_19D | 2035 | Orchis | Orchis militaris |  |
| 43 | Heinrich, Marstaller & Voigt (2012) | CAT_20A | 2038 | Platanthera | Platanthera chlorantha |  |
| 43 | Heinrich, Marstaller & Voigt (2012) | CAT_20C | 2040 | Orchis | Orchis militaris |  |
| 43 | Heinrich, Marstaller & Voigt (2012) | CAT_20D | 2041 | Orchis | Orchis militaris |  |
| 43 | Heinrich, Marstaller & Voigt (2012) | CAT_21B | 2045 | Orchis | Orchis x hybrida |  |
| 43 | Heinrich, Marstaller & Voigt (2012) | CAT_21C | 2046 | Orchis | Orchis purpurea |  |
| 43 | Heinrich, Marstaller & Voigt (2012) | CAT_22A | 2050 | Orchis | Orchis purpurea |  |
| 43 | Heinrich, Marstaller & Voigt (2012) | CAT_22B | 2051 | Platanthera | Platanthera chlorantha |  |
| 43 | Heinrich, Marstaller & Voigt (2012) | CAT_22E | 2054 | Orchis | Orchis militaris |  |
| 43 | Heinrich, Marstaller & Voigt (2012) | CAT_23A | 2056 | Orchis | Orchis purpurea |  |
| 43 | Heinrich, Marstaller & Voigt (2012) | CAT_23B | 2057 | Platanthera | Platanthera chlorantha |  |
| 43 | Heinrich, Marstaller & Voigt (2012) | CAT_28A | 2086 | Orchis | Orchis militaris |  |
| 43 | Heinrich, Marstaller & Voigt (2012) | M_CF3 | 471 | Ulmus | Ulmus glabra |  |
| 43 | Heinrich, Marstaller & Voigt (2012) | M_CF3 | 495 | Ulmus | Ulmus glabra |  |
| 43 | Heinrich, Marstaller & Voigt (2012) | M_CF3 | 519 | Ulmus | Ulmus glabra |  |
| 43 | Heinrich, Marstaller & Voigt (2012) | M_CF4 | 568 | Orchis | Orchis x hybrida |  |
| 43 | Heinrich, Marstaller & Voigt (2012) | M_CF6 | 570 | Orchis | Orchis militaris |  |
| 43 | Horchler (unpubl.) | 67 | 93 | Callitriche | Callitriche palustris agg. |  |
| 43 | Horchler (unpubl.) | 68 | 94 | Callitriche | Callitriche palustris agg. |  |
| 43 | Hüllbusch et al. (2016) | MW9 | 28 | Tragopogon | Tragopogon pratensis |  |
| 43 | Kuhn et al. (2011) | 4350724.00_5487257.00 | 1608 | Campanula | Campanula rapunculoides |  |
| 74 | Kuhn et al. (2011) | 4365134.20_5531963.60 | 1300 | Primula | Primula veris |  |
| 74 | Kuhn et al. (2011) | 4365183.00_5532063.00 | 1301 | Campanula | Campanula rapunculoides |  |
| 19 | Kuhn et al. (2011) | 4365308.00_5532308.00 | 1302 | Campanula | Campanula rapunculoides |  |
| 14 | Kuhn et al. (2011) | 4377860.10_5547643.00 | 1357 | Primula | Primula veris |  |
| 14 | Kuhn et al. (2011) | 4395362.00_5271657.00 | 2024 | Primula | Primula elatior |  |
| 14 | Kuhn et al. (2011) | 4415784.00_5532303.00 | 832 | Primula | Primula veris |  |
| 14 | Kuhn et al. (2011) | 4420009.00_5433917.00 | 3580 | Agrimonia | Agrimonia eupatoria |  |
| 14 | Kuhn et al. (2011) | 4421750.00_5573096.00 | 34 | Ajuga | Ajuga reptans |  |
| 14 | Kuhn et al. (2011) | 4450612.00_5509207.00 | 769 | Ajuga | Ajuga reptans |  |
| 14 | Meineke & Menge (2010) | DBF 4 | 16 | Alchemilla | Alchemilla vulgaris agg. |  |
| 14 | Meineke & Menge (2010) | DBF 4 | 17 | Alchemilla | Alchemilla vulgaris agg. |  |
| 14 | Meineke & Menge (2010) | DBF 4 | 18 | Alchemilla | Alchemilla vulgaris agg. |  |
| 14 | Meineke & Menge (2010) | DBF 4 | 19 | Alchemilla | Alchemilla vulgaris agg. |  |
| 55 | Meineke & Menge (2010) | DBF 8 | 36 | Alchemilla | Alchemilla vulgaris agg. |  |
| 55 | Meineke & Menge (2010) | DBF 8 | 37 | Alchemilla | Alchemilla vulgaris agg. |  |
| 55 | Meineke & Menge (2010) | DBF 8 | 38 | Alchemilla | Alchemilla vulgaris agg. |  |
| 55 | Meineke & Menge (2010) | DBF 8 | 40 | Alchemilla | Alchemilla vulgaris agg. |  |
| 55 | Meineke & Menge (2010) | DBF 10 | 46 | Alchemilla | Alchemilla vulgaris agg. |  |
| 55 | Meineke & Menge (2010) | DBF 10 | 47 | Alchemilla | Alchemilla vulgaris agg. |  |
| 55 | Meineke & Menge (2010) | DBF 10 | 48 | Alchemilla | Alchemilla vulgaris agg. |  |
| 55 | Meineke & Menge (2010) | DBF 10 | 50 | Alchemilla | Alchemilla vulgaris agg. |  |
| 55 | Meineke & Menge (2010) | DBF 11 | 51 | Alchemilla | Alchemilla vulgaris agg. |  |
| 55 | Meineke & Menge (2010) | DBF 11 | 52 | Alchemilla | Alchemilla vulgaris agg. |  |
| 55 | Meineke & Menge (2010) | DBF 11 | 53 | Alchemilla | Alchemilla vulgaris agg. |  |
| 55 | Meineke & Menge (2010) | DBF 11 | 55 | Alchemilla | Alchemilla vulgaris agg. |  |
| 55 | Meineke & Menge (2010) | DBF 13 | 61 | Alchemilla | Alchemilla vulgaris agg. |  |
| 55 | Meineke & Menge (2010) | DBF 13 | 62 | Alchemilla | Alchemilla vulgaris agg. |  |
| 55 | Meineke & Menge (2010) | DBF 13 | 63 | Alchemilla | Alchemilla vulgaris agg. |  |
| 55 | Meineke & Menge (2010) | DBF 13 | 65 | Alchemilla | Alchemilla vulgaris agg. |  |
| 55 | Müller (2002) | K/SH1 | 119 | Thymus | Thymus praecox agg. |  |
| 55 | Peppler-Lisbach & Könitz (2017) | N 86-192 | 56 | Euphorbia stricta | Euphrasia stricta |  |
| 55 | Peppler-Lisbach & Könitz (2017) | N 86-193 | 57 | Euphorbia stricta | Euphrasia stricta |  |
| 2 | P | B | 1 | P | P |  |
| 3 | P | B | 1 | P | P | P |
| 3 | P | B | 1 | P | P |  |
| 32 | Raehse (2001) | M546/50 | 880 | Fragaria | Fragaria vesca |  |
| 44 | Schmidt et al. (unpubl.) | Hünstollen_KF1_37 | 1825 | Anemone | Anemone nemorosa |  |
| 44 | Schmidt et al. (unpubl.) | Hünstollen_KF1_40 | 1828 | Anemone | Anemone nemorosa |  |
| 44 | Schmidt et al. (unpubl.) | Hünstollen_KF1_41 | 1829 | Anemone | Anemone nemorosa | Anemone ranunculoides |
| 36 | Schmidt et al. (unpubl.) | Hünstollen_KF1_42 | 1830 | Anemone | Anemone nemorosa | Anemone ranunculoides |
| 33 | Schmidt et al. (unpubl.) | Hünstollen_KF1_43 | 1831 | Anemone | Anemone nemorosa |  |
| 33 | Schmidt et al. (unpubl.) | Hünstollen_KF1_44 | 1832 | Anemone | Anemone nemorosa |  |
| 33 | Schmidt et al. (unpubl.) | Hünstollen_KF1_45 | 1833 | Anemone | Anemone nemorosa |  |
| 33 | Schmidt et al. (unpubl.) | Hünstollen_KF1_46 | 1834 | Anemone | Anemone nemorosa |  |
| 33 | Schmidt et al. (unpubl.) | Hünstollen_KF1_47 | 1835 | Anemone | Anemone nemorosa |  |
| 33 | Schmidt et al. (unpubl.) | Hünstollen_KF1_48 | 1836 | Anemone | Anemone nemorosa | Anemone ranunculoides |
| 33 | Schmidt et al. (unpubl.) | Hünstollen_KF1_49 | 1837 | Anemone | Anemone nemorosa |  |
| 33 | Schmidt et al. (unpubl.) | Hünstollen_KF1_50 | 1838 | Anemone | Anemone nemorosa |  |
| 33 | Schmidt et al. (unpubl.) | Hünstollen_KF1_51 | 1839 | Anemone | Anemone nemorosa |  |
| 33 | Schmidt et al. (unpubl.) | Hünstollen_KF2_30 | 1869 | Anemone | Anemone nemorosa | Anemone ranunculoides |
| 33 | Schmidt et al. (unpubl.) | Hünstollen_KF2_32 | 1871 | Anemone | Anemone nemorosa | Anemone ranunculoides |
| 33 | Schmidt et al. (unpubl.) | Hünstollen_KF2_34 | 1873 | Anemone | Anemone nemorosa | Anemone ranunculoides |
| 33 | Schmidt et al. (unpubl.) | Hünstollen_KF2_35 | 1874 | Anemone | Anemone nemorosa | Anemone ranunculoides |
| 33 | Schmidt et al. (unpubl.) | Hünstollen_KF2_37 | 1876 | Anemone | Anemone nemorosa | Anemone ranunculoides |
| 33 | Schmidt et al. (unpubl.) | Hünstollen_KF2_41 | 1880 | Anemone | Anemone nemorosa |  |
| 33 | Schmidt et al. (unpubl.) | Hünstollen_KF2_42 | 1881 | Anemone | Anemone nemorosa |  |
| 33 | Schmidt et al. (unpubl.) | Hünstollen_KF2_43 | 1882 | Anemone | Anemone nemorosa |  |
| 33 | Schmidt et al. (unpubl.) | Hünstollen_KF2_44 | 1883 | Anemone | Anemone nemorosa |  |
| 33 | Strubelt & Zacharias (2015) | 8 | 41 | Campanula | Campanula trachelium |  |
